# A Cell-Free Fluorometric High-Throughput Screen for Inhibitors of Rtt109-Catalyzed Histone Acetylation

**DOI:** 10.1371/journal.pone.0078877

**Published:** 2013-11-18

**Authors:** Jayme L. Dahlin, Rondedrick Sinville, Jonathan Solberg, Hui Zhou, Junhong Han, Subhashree Francis, Jessica M. Strasser, Kristen John, Derek J. Hook, Michael A. Walters, Zhiguo Zhang

**Affiliations:** 1 Department of Molecular Pharmacology and Experimental Therapeutics, Mayo Clinic College of Medicine, Rochester, Minnesota, United States of America; 2 Medical Scientist Training Program, Mayo Clinic College of Medicine, Rochester, Minnesota, United States of America; 3 Institute for Therapeutics Discovery & Development, University of Minnesota, Minneapolis, Minnesota, United States of America; 4 Department of Biochemistry and Molecular Biology, Mayo Clinic College of Medicine, Rochester, Minnesota, United States of America; The Walter and Eliza Hall of Medical Research, Australia

## Abstract

The lysine acetyltransferase (KAT) Rtt109 forms a complex with Vps75 and catalyzes the acetylation of histone H3 lysine 56 (H3K56ac) in the Asf1-H3-H4 complex. Rtt109 and H3K56ac are vital for replication-coupled nucleosome assembly and genotoxic resistance in yeast and pathogenic fungal species such as *Candida albicans*. Remarkably, sequence homologs of Rtt109 are absent in humans. Therefore, inhibitors of Rtt109 are hypothesized as potential and minimally toxic antifungal agents. Herein, we report the development and optimization of a cell-free fluorometric high-throughput screen (HTS) for small-molecule inhibitors of Rtt109-catalyzed histone acetylation. The KAT component of the assay consists of the yeast Rtt109-Vps75 complex, while the histone substrate complex consists of full-length *Drosophila* histone H3-H4 bound to yeast Asf1. Duplicated assay runs of the LOPAC demonstrated day-to-day and plate-to-plate reproducibility. Approximately 225,000 compounds were assayed in a 384-well plate format with an average Z' factor of 0.71. Based on a 3σ cut-off criterion, 1,587 actives (0.7%) were identified in the primary screen. The assay method is capable of identifying previously reported KAT inhibitors such as garcinol. We also observed several prominent active classes of pan-assay interference compounds such as Mannich bases, catechols and p-hydroxyarylsulfonamides. The majority of the primary active compounds showed assay signal interference, though most assay artifacts can be efficiently removed by a series of straightforward counter-screens and orthogonal assays. Post-HTS triage demonstrated a comparatively small number of confirmed actives with IC_50_ values in the low micromolar range. This assay, which utilizes five label-free proteins involved in H3K56 acetylation *in vivo*, can in principle identify compounds that inhibit Rtt109-catalyzed H3K56 acetylation via different mechanisms. Compounds discovered via this assay or adaptations thereof could serve as chemical probes or leads for a new class of antifungals targeting an epigenetic enzyme.

## Introduction

Eukaryotic DNA is assembled into chromatin, the repeating structure of which is known as the nucleosome. Chromatin encodes epigenetic information and is important in regulating gene transcription, DNA repair and replication. Chromatin is subject to regulation by post-translational modifications such as methylation, acetylation, phosphorylation and ubiquitylation of histones, which are the protein structural component of the nucleosome. Histone acetylation can alter chromatin structure and also recruit additional protein machinery involved in chromatin regulation [1], [2]. Histone acetylation is catalyzed by lysine acetyltransferases (KATs), which catalyze the transfer of an acetyl group from acetyl-CoA to ε-amino groups on specific histone lysine residues. One specific and atypical modification first discovered in *Saccharomyces cerevisiae*, histone H3 lysine 56 acetylation (H3K56ac), occurs during the S phase of the cell cycle and is catalyzed by the KAT protein Rtt109 [3]–[6]. Rtt109-catalyzed H3 acetylation promotes genotoxin survival due in part to its role in DNA synthesis-dependent nucleosome assembly associated with DNA replication and DNA repair [5]–[8]. In humans, H3K56ac is catalyzed by p300/CBP or GCN5 [9], [10], of which the catalytic motif of p300 shares tertiary structural similarity with Rtt109 despite possessing little primary sequence homology [11], [12].

The catalytic activity of Rtt109 is subject to complex regulation. In the absence of autoacetylation [13], [14] or the chaperone proteins Asf1 or Vps75, *S. cerevisiae* Rtt109 (scRtt109) has lower KAT activity [4], [6], [15], [16]. Vps75 is a member of the NAP1 histone chaperone family and forms a stable complex with Rtt109 *in vivo* and *in vitro*. While the Rtt109-Vps75 complex is capable of acetylating H3K56, Vps75 is not essential for H3K56 acetylation *in vivo* [4], [17]–[20]. Vps75 also enhances Rtt109-catalyzed acetylation of H3K4, H3K9, H3K14, H3K18, H3K23 and H3K27 *in vitro* [20]–[23]. *In vivo*, the histone chaperone Asf1 is also essential for H3K56ac, and it dramatically enhances the activity of the Rtt109-Vps75 complex towards H3K56ac. However, it remains to be determined whether Asf1 is also required for Rtt109-catalyzed acetylation of lysine residues at the H3 N-terminus *in vivo*. Nonetheless, it is likely that Rtt109-Vps75 utilizes Asf1-H3-H4 as the substrate *in vivo* for at least H3K56 acetylation.

Opportunistic fungal infections can severely compromise the therapeutic outcome of cancer patients, organ transplant patients and other immunocompromised patients. The crude mortality rate from opportunistic fungal infection exceeds 50% in many human studies [24]–[26]. Fungi are difficult to treat therapeutically because of several factors. First, fungi are eukaryotes, and many of their biologically crucial genes are also conserved in humans. Therefore, it has proven difficult to find fungi-specific therapeutic targets that minimize toxicity to humans [27]. Second, fungi can develop resistance to most drugs currently used to treat patients [28]. Third, early detection and diagnosis of fungal infections can be difficult in clinical settings [29], [30]. Finally, fungal pathogenesis is governed by complicated host-pathogen interactions [31], [32]. Therefore, there is a clinical need for novel and efficacious antifungal treatments.

Inhibiting Rtt109-catalyzed histone acetylation may be clinically relevant for antifungal purposes. While Rtt109 is highly conserved in fungal species, it exhibits no obvious sequence homology to mammalian KATs. Additionally, it appears that Rtt109 utilizes a different catalytic mechanism than p300/CBP [33], the potential functional homolog of Rtt109. Inhibitors of p300 such as Lys-CoA have not shown activity versus Rtt109 [11]. Furthermore, others have shown that deletion of *rtt109* in *Candida albicans* reduces fungal virulence in mouse models [34], [35]. Our group has also shown *Pneumocystis carinii* expresses an active Rtt109 KAT [36], [37]. These results support the idea that Rtt109 is an attractive antifungal therapeutic target and that compounds that inhibit Rtt109-catalyzed histone acetylation may serve as potential antifungal agents. To date, only one small-molecule has been reported to inhibit Rtt109-catalyzed histone acetylation but not other KATs like GCN5 and p300. This particular molecule did not affect cellular levels of H3K56ac or sensitivity to a genotoxic agent in either *C. albicans* or *S. cerevisiae* [38], but this lack of observed *in vivo* activity could be due to a variety of factors such as drug metabolism, cell permeability or degradation. Therefore, methods must still be developed and optimized to identify compounds capable of inhibiting Rtt109-catalyzed histone acetylation, both *in vitro* and *in vivo*.

In this work, we report the development and optimization of a high-throughput screen (HTS) for small-molecule inhibitors of Rtt109-catalyzed histone acetylation using the well-characterized *S. cerevisiae* Rtt109–Vps75-Asf1 proteins. The assay quantifies the amount of free coenzyme A (CoA), a by-product of the Rtt109-catalyzed KAT reaction, by use of the thiol-sensitive probe 7-diethylamino-3-(4′-maleimidyl-phenyl)-4-methylcoumarin (CPM) [39], and is an adaption of previous CPM-based assays [40]–[43]. Importantly, our assays take into account the complex regulation of Rtt109 *in vitro* and *in vivo* by using purified Rtt109-Vps75 as the enzyme and Asf1 bound to full-length H3-H4 as the substrate. Given the susceptibility of this assay format to false-positives, appropriate follow-up assays and consideration of assay-specific artifacts and promiscuous inhibitors are also discussed herein.

## Materials and Methods

### Molecular libraries, compounds and reagents

The chemical library consisted of a 100,000-compound library (ChemBridge), the 1,280-member Library of Pharmacologically Active Compounds (LOPAC; Sigma-Aldrich), 100,000 compounds acquired from the University of Kansas High-Throughput Screening Laboratory [44], focused natural product libraries and also 6,700 peptide and peptidomimetic compounds. Additional compounds were obtained from the MicroSource Spectrum Collection (Discovery Systems), the Prestwick Chemical collection, the NIH Clinical Collection, Tocris chemical standard libraries (Tocris Bioscience), in-house and miscellaneous commercial compounds.

The following chemicals and reagents were obtained from Sigma: DMSO, CPM, coenzyme A (sodium salt hydrate), acetyl-CoA (sodium salt), aurintricarboxylic acid (ATA), Triton X-100, L-glutathione, fluconazole, lidocaine, rottlerin, NSC-663284, Horseradish peroxidase and phenol red. The following compounds were also obtained commercially: 4-amino-1-naphthol (TCI America) and nitrocefin (TOKU-E). Garcinol (Enzo Life Sciences) was purchased and used without additional purification. Compound **1** and the compounds evaluated for IC_50_ values were obtained from commercial sources and tested without additional purification. Garcinol and compound **1** showed greater than 98% purity by UPLC/MS analyses. The ^1^H and ^13^C NMR spectra for garcinol and compound **1** were consistent with their reported chemical structures. The ^1^H NMR (400 MHz) and ^13^C NMR (100 MHz) spectra were recorded on a Bruker Avance spectrometer, while the UPLC/MS analyses were performed using a Waters Acquity UPLC with ZQ mass spectrometer.

### Protein production and purification

*Drosophila* H3-H4 (dH3-H4) was obtained as previously described [45]. Purified *S. cerevisiae* Asf1 was incubated with approximately equal molar amounts of purified dH3-H4 overnight at 4°C, and the resulting Asf1-dH3-H4 complex was then purified by gel filtration chromatography in the absence of dithiothreitol (DTT) [16], as DTT interferes with the CPM thiol probe. The *S. cerevisiae* Rtt109-Vps75 complex was prepared as previously described, except that residual DTT was removed through gel filtration in the final purification step [46]. Protein concentrations were determined by the Bradford method [47]. Purified protein complexes were stored at −80°C in their appropriate buffered solutions plus 10% glycerol (v/v) until the day of use.

### High-throughput screen in 384-well plate format

A standardized description of each assay step is provided (Tables 1 and 2) [48]. Briefly, test compounds and the control inhibitor ATA were first added to the appropriate wells using an Echo 550 contactless liquid handler (Labcyte). Test compounds were prepared as 10 mM stock solutions dissolved in DMSO, stored at −20°C under vacuum seals and screened at 10 µM final compound concentrations. Buffer, purified Rtt109-Vps75 and purified Asf1-dH3-H4 substrate were added in sequential dispensing steps. Compounds and proteins were then allowed to incubate for 15 min at 30°C to allow for equilibration. The KAT reaction was then initiated by dispensing 10 µL acetyl-CoA to appropriate wells to yield a final reaction concentration of 10 µM acetyl-CoA and 15 µL total solution volume per well. Each tested microplate had negative control wells (acetyl-CoA+DMSO) and positive control wells (Rtt109-Vps75+Asf1-dH3-H4+acetyl-CoA+DMSO), as well as compound control wells (Rtt109-Vps75+Asf1-dH3-H4+acetyl-CoA+10 µM ATA). Plates were sealed using a PlateLoc apparatus (Agilent). The reactions were allowed to proceed for up to 45 min at 30°C. The extent of CoA production was then assessed by dispensing 5 µL of an 80 µM CPM solution to each well, yielding a final CPM concentration of 20 µM. After a short incubation period, microplates were read on a fluorescent plate reader. For the second HTS production run (HTS2), the buffers also contained freshly prepared 0.01% Triton X-100 (v/v) [49].

**Table 1:** Rtt109-Vps75 HTS protocol steps.

Step	Parameter	Value	Description
1	Compound controls, DMSO	15 nL	ATA to wells A1-H1 and I23-P23; DMSO to wells I1-P1, A23-H23 and columns 2 and 24
2	Test compounds	15 nL	Compounds to wells A3-P22
3	Buffer	5 µL	KAT buffer to columns 2 and 24
4	Enzyme complex	2.5 µL	Rtt109-Vps75 to columns 1 and 3–23
5	Protein substrate	2.5 µL	Asf1-dH3-H4 to columns 1 and 3–23
6	Incubation time	15 min	30°C
7	Acetyl-CoA	10 µL	Acetyl-CoA to all wells; final well volume = 15 µL
8	Plate shake time	5 min	Mix well contents
9	Incubation time	45 min	30°C
10	CPM probe	5 µL	CPM to all wells; final well volume = 20 µL
11	Plate shake time	1 min	Mix plate contents
12	Assay readout	405 and 530 nm	Measure fluorescence intensity (λ_ex_, λ_em_, respectively)

**Table 2:** Rtt109-Vps75 HTS protocol notes.

Step	Notes
1	ATA (10 mM DMSO stock solution, freshly prepared), 10 µM final reaction concentration; transfer to assay plates with Echo 550 contactless liquid handler
2	Compounds (10 mM DMSO stock solutions), 10 µM final reaction concentration; transfer to plates with Echo 550
3	KAT buffer (50 mM Tris-HCl, pH 8.0, 50 mM KCl, 0.1 mM EDTA, ±0.01% Triton X-100 (v/v)); transfer to plates using Thermo MultiDrop; Corning 384-well black polystyrene, low-volume, non-treated microplates (model #3677)
4	200 ng Rtt109-Vps75 complex per well (80 ng/µL dispensing solution, dissolved in KAT buffer); transfer to plates with MultiDrop
5	800 ng Asf1-dH3-H4 complex per well (320 ng/µL dispensing solution, dissolved in KAT buffer); transfer to plates with MultiDrop
7	Acetyl-CoA (freshly prepared; 10 mM H_2_O stock solution dissolved in KAT buffer to make 15 µM dispensing solution); 10 µM final reaction concentration; transfer to plates with MultiDrop
8	DPC micromix shaker
9	Seal plate with PlateLoc
10	Briefly centrifuge and then unseal plates; CPM (freshly prepared 50 mM DMSO stock solution, then diluted with 1∶1 EtOH∶H_2_O (v/v) to make 80 µM dispensing solution); final concentration will be 20 µM; transfer to plates with MultiDrop
11	Micromix shaker; then allow 10 min at room temperature for reaction to complete
12	SpectraMax M2E plate reader (Molecular Devices)

### Dose-response and IC_50_ experiments

Compounds selected for dose-response analyses were tested in duplicate at eight compound concentrations, ranging from 40 nM to 125 µM final compound concentrations. Plate controls, dispensing steps and general assay procedures were unchanged from the aforementioned HTS procedures. Slot blots were performed using standard techniques with a Bio-Rad Bio-Dot SF microfiltration apparatus. Membranes were imaged with a LI-COR Odyssey and analyzed using Image Studio (LI-COR Biosciences). For the slot blot experiments, the assay plates included an additional plate control consisting of the Rtt109-Vps75 and Asf1-dH3-H4 complexes and no acetyl-CoA. Dose-response and slot blot experiments were replicated at least three times by independent experiments. IC_50_ values on purchased samples were determined as described above, except compounds were tested in triplicate at twelve compound concentrations, ranging from 40 nM to 250 µM final compound concentrations.

### Data analysis

Z' factors for each plate were calculated in ActivityBase (IDBS) using Equation (1) [50]:
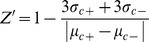
(1)where σ and μ represent the standard deviation and mean of the positive (c+) and negative (c−) -plate controls, respectively. Plates with Z' factors below 0.4 were re-screened. The percent inhibitions of test compounds were calculated based on the positive and negative controls on each plate using ActivityBase. Dose-response curves were generated in GraphPad Prism 6.0 using the sigmoidal dose-response variable slope four-parameter Equation (2):
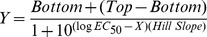
(2)Lines of best fit were determined by linear regression in Prism. Unless noted otherwise, standard deviations are indicated after mean values and depicted as error bars.

### Cheminformatics

Pan-assay interference compounds (PAINS) were identified by substructure query [51] using SYBYL-X (Tripos) on a Linux workstation. The computational triage of primary HTS active compounds was also performed in the SYBYL-X workspace using the built-in HTS filter and custom substructure filters.

### Fluorescence quenching counter-screen

CPM and CoA (20 and 5 µM final concentrations, respectively) were allowed to react to completion in HTS buffer, as assessed by reading the fluorescence intensity at 5 min intervals until a stable signal plateau was observed. The resulting CPM-CoA adduct solutions were then spiked with either DMSO or test compounds using a contactless liquid dispenser. The microplates were shaken for 5 min and allowed to equilibrate for another 5 min at room temperature. The fluorescence intensity was then measured, and the data was analyzed as percent signal reduction compared to DMSO controls.

### Assay interference counter-screen

Compounds interfering with the CPM-based assay readout were identified using an adaption of published procedures [41], [52]. Selected compounds were tested in duplicate at 10 and 20 µM final concentrations using the HTS assay format, except proteins were absent and the acetyl-CoA substrate was replaced with the CoA by-product in concentrations that were titred to the fluorescence intensity observed at approximately zero and 50% inhibition. Compounds were incubated with CoA under conditions identical to the HTS, after which CPM was added as described previously. Assay interference was quantified by comparing the (compound+CoA+CPM) fluorescence intensities to the (DMSO+CoA+CPM) controls. All plates contained background controls (DMSO+CoA) and (DMSO+CPM). Triage criteria included a combination of significant signal interference (generally greater than 50% control) in at least one tested condition, interference reproducibility, and evidence of CoA- and/or compound-dependent interference.

### Compound-thiol adduct assay

High micromolar concentrations of selected compounds (1 equiv) and either CoA or L-glutathione (1–4 equiv) were incubated under HTS-like conditions. Detergent was excluded from the sample buffers. CPM was used as a positive adduct control, as well as several in-house compounds that form known thiol adducts by several different chemical mechanisms under the HTS conditions. The samples were analyzed on a Waters UPLC system using a BEH C18 2.1×50 mm column. The flow rate was 0.25 mL/min with a standard gradient starting at 95% Solution A (950 mL H_2_O, 50 mL MeCN, 1 mL formic acid) and ending with 100% Solution B (1000 mL MeCN, 1 mL formic acid) over 6.5 min. The samples were monitored simultaneously using an ELS detector, a diode array detector (214, 220, 244 and 254 nm) and a ZQ mass spectrometer (ESI positive and negative modes).

### Redox-activity and aggregation triage

Selected compounds were de-risked for redox-activity using a published protocol [53] except that the published assay buffer was swapped for the Rtt109 HTS assay buffer. 100 µM H_2_O_2_ was used as a positive plate control, while NSC-663284 and 4-amino-1-naphthol were used as positive redox-active controls for DTT and DTT-free assay conditions, respectively [53], [54]. Fluconazole and DMSO were used as negative compound and plate controls, respectively. Compounds were tested in duplicate at eight final concentrations (1.25 to 160 µM via two-fold dilutions) in either the presence or absence of 1 mM DTT final concentration. Selected compounds were also de-risked for aggregation behavior using published procedures [55], except that the published assay buffer was again swapped for the HTS buffer. Rotterlin and lidocaine were used as a positive and negative compound controls, respectively [56]. Compounds were tested at 10 µM final concentrations in either the presence or absence of 0.01% Triton X-100.

### [^3^H]-acetyl CoA orthogonal assay

Selected compounds were tested for inhibition of KAT activity using an alternative *in vitro* orthogonal assay employing [^3^H]-acetyl-CoA. Inhibition of KAT activity was measured at up to ten compound concentrations (40 nM to 250 µM final compound concentrations) as previously reported [57], with some minor modifications. Briefly, reactions were performed in 60 µL volumes containing the following in final concentrations: 50 mM Tris HCl, pH 8.0, 50 mM KCl, 0.1 mM EDTA, 1 mM DTT, 0.01% Triton X-100 (v/v) and 2.5 µM [^3^H]-acetyl-CoA (Perkin Elmer). Purified Rtt109-Vps75 was tested at approximately 5 nM final concentrations, while purified Asf1-dH3-H4 (approximately 250 nM) was used as acetylation substrate. Test compounds were allowed to pre-equilibrate with enzyme and histone substrate for 10 min at 30°C before initiating the KAT reaction with the addition of [^3^H]-acetyl-CoA. DMSO content was kept constant at 3% (v/v). The KAT reactions were allowed to proceed for 5 min, after which aliquots (20 µL) were immediately spotted onto Whatman phosphocellulose paper filters (GE Healthcare) and air-dried. Filter papers were washed five times for 5 min per cycle with 50 mM NaHCO_3_, pH 9.0, and rinsed with acetone and then allowed to air-dry for 30 min. [^3^H]-acetate incorporation was then measured by an LS6500 liquid scintillation counter (Beckman-Coulter). Percent inhibition was calculated as a percentage of DMSO control.

## Results

### HTS development and optimization

**Figure 1:**
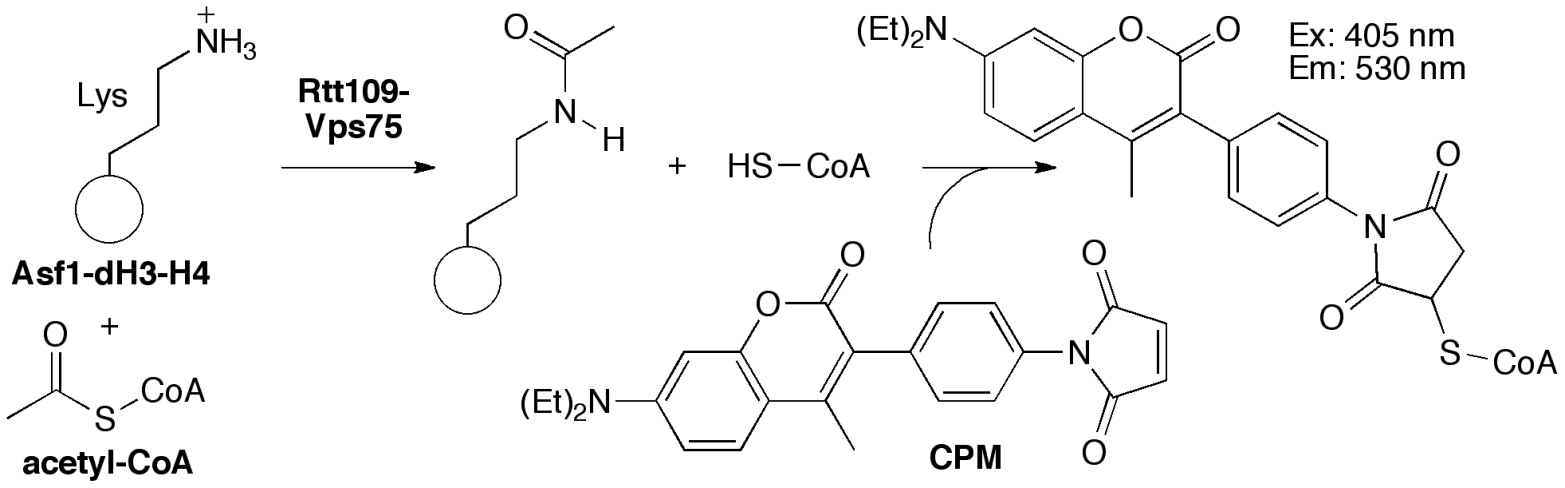
Fluorometric Rtt109-Vps75 HTS schematic. The Rtt109-Vps75 KAT complex catalyzes the transfer of an acetyl group from acetyl-CoA to specific histone lysine residues within the Asf1-dH3-H4 substrate complex. The resulting CoA contains a free thiol group (-SH), which can react with the sulfhydryl-sensitive probe CPM to form a fluorescent adduct, thereby permitting the quantification of free CoA as a measure of KAT activity.

We developed a cell-free 384-well plate assay for Rtt109-catalyzed histone acetylation based on previously reported assays probing for the presence of CoA [40], [41]. The purified Rtt109-Vps75 complex catalyzes the transfer of an acetyl group from acetyl-CoA to the ε-amino group on specific lysine residues on histone H3, which is part of the substrate complex consisting of purified full-length histone H3-H4 tetramers bound to the chaperone protein Asf1. The byproduct of this reaction, free coenzyme A (represented as either CoA or CoA-SH to denote the free thiol group), accumulates with increasing KAT activity. At the assay endpoint, CoA is quantified by the addition of CPM, which readily reacts with available CoA-SH to produce a fluorescent thioester adduct through a Michael addition with the maleimide moiety on CPM (Figure 1).

**Figure 2:**
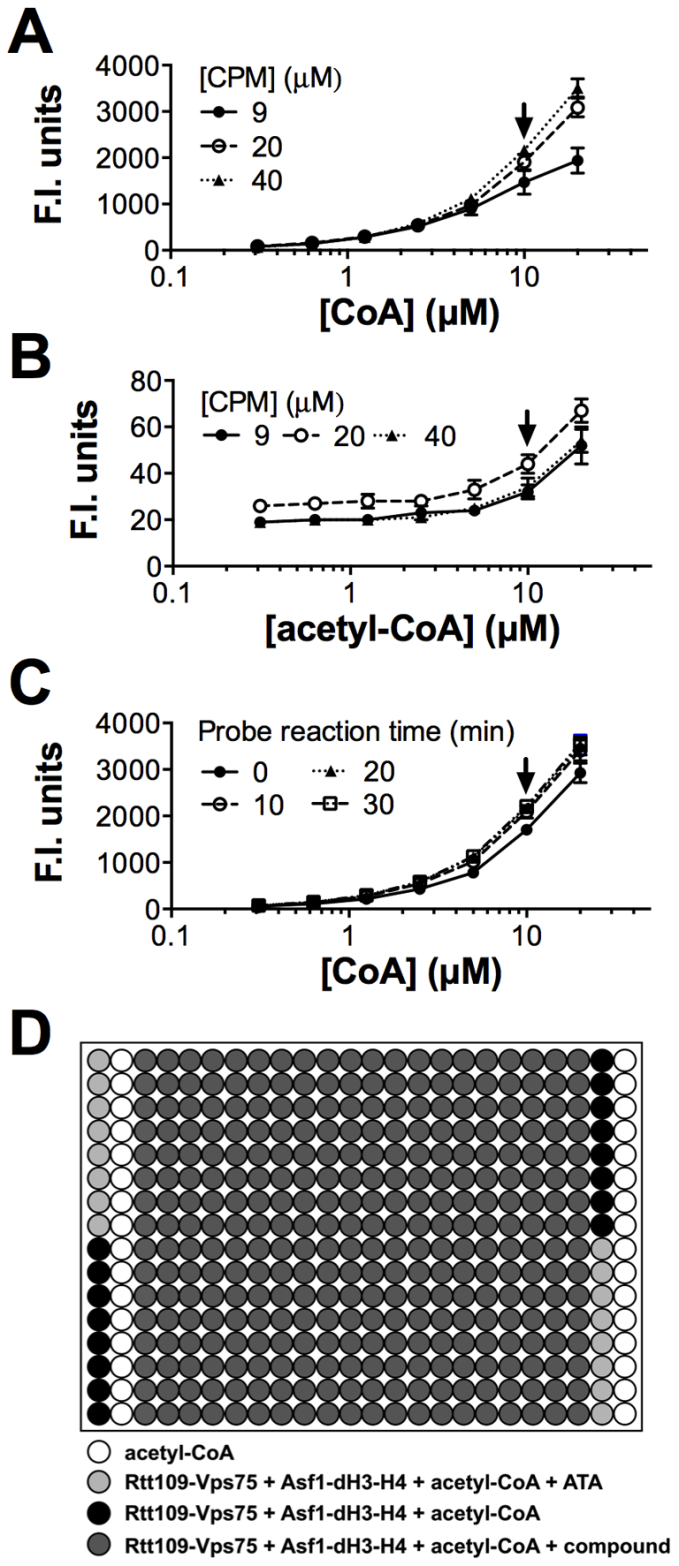
Assay design and optimization. (A) Titration matrix of CoA and CPM in buffer-only conditions to determine the optimal assay levels of acetyl-CoA and CPM. (B) Titration matrix of CPM and acetyl-CoA in buffer-only conditions to verify acetyl-CoA and CPM do not form fluorescent adducts under HTS conditions. (C) Time-course study of CoA titrations with 20 µM CPM in buffer-only conditions to determine the optimal time for the reaction involving CoA and CPM. (D) HTS plate template. Arrows denote chosen HTS conditions.

Several experiments were performed to optimize the reaction conditions for an HTS-compatible format. Rtt109 is a complicated bi-substrate enzymatic system [33], which inevitably poses more challenges with regards to assay optimization than a mono-substrate system [58]. As was done in a previous screen for histone acetyltransferases [59], we chose 10 µM as the amount of acetyl-CoA, which is close to a previous report of the apparent Michaelis-Menten constant for Rtt109-Vps75 (8 µM) [11]. Titrations of the Rtt109-Vps75 and Asf1-dH3-H4 complexes were performed to determine the optimal amounts of each protein complex per well. To maximize signal intensity and yet minimize the amount of protein complexes needed for a large-scale HTS, 200 ng Rtt109-Vps75 (approximately 120 nM) and 800 ng Asf1-dH3-H4 (approximately 920 nM) per well were chosen as protein concentrations (data not shown). Next, the optimal balance of CPM and acetyl-CoA were determined in order to achieve a sufficient signal∶background ratio. Based on this titration matrix, subsequent experiments used CPM and acetyl-CoA in final concentrations of 20 and 10 µM, respectively (Figure 2A). We also confirmed that acetyl-CoA did not produce appreciable fluorescent signals in the presence of CPM in our assay conditions (Figure 2B). The reaction between 20 µM CPM and CoA titrations was relatively rapid, as indicated by the signal plateau by 10 min reaction time (Figure 2C). This finding is consistent with a recent report on the reaction kinetics of CPM [60] and therefore subsequent experiments allowed CPM to react for up to 10 min before data acquisition. A low-volume microplate allowed a more intense signal and a moderately improved signal∶background ratio compared to a standard-volume microplate using identical reaction volumes and components (data not shown). We also verified by time-course monitoring that the KAT reaction was not complete at the assay endpoint (data not shown) [58]. The 384-well plate layout includes positive control wells with no compounds present, negative control wells with no proteins present and also compound control wells with ATA (corresponding to 50% signal reduction at 10 µM final concentration) on the plate edges (Figure 2D). For each 384-well plate, 320 compounds were tested at a single concentration.

### Division of HTS into two production runs

The optimized assay screened 225,703 compounds in two separate, non-overlapping production runs, hereby referred to as HTS1 and HTS2. The first production run (HTS1) was performed with a detergent-free buffer. After screening approximately one-third of the chemical library, the number of compounds showing apparent inhibition combined with a preliminary analysis of the chemical nature of the active compounds suggested some chemical aggregators were also being identified as actives in the primary assay [61], [62]. Based on these observations, the second production run (HTS2) included 0.01% Triton X-100 to reduce the odds of identifying additional non-specific chemical aggregators [56], [62]–[64]. Inclusion of the detergent did not adversely affect enzyme performance (data not shown). The 82,861 compounds screened in HTS1 under detergent-free conditions were not re-screened in the presence of detergent, as we opted instead to analyze the two production runs separately and perform future follow-up experiments of HTS1 compounds in the presence of detergent-containing buffers.

### Assay evaluation using the LOPAC

**Figure 3:**
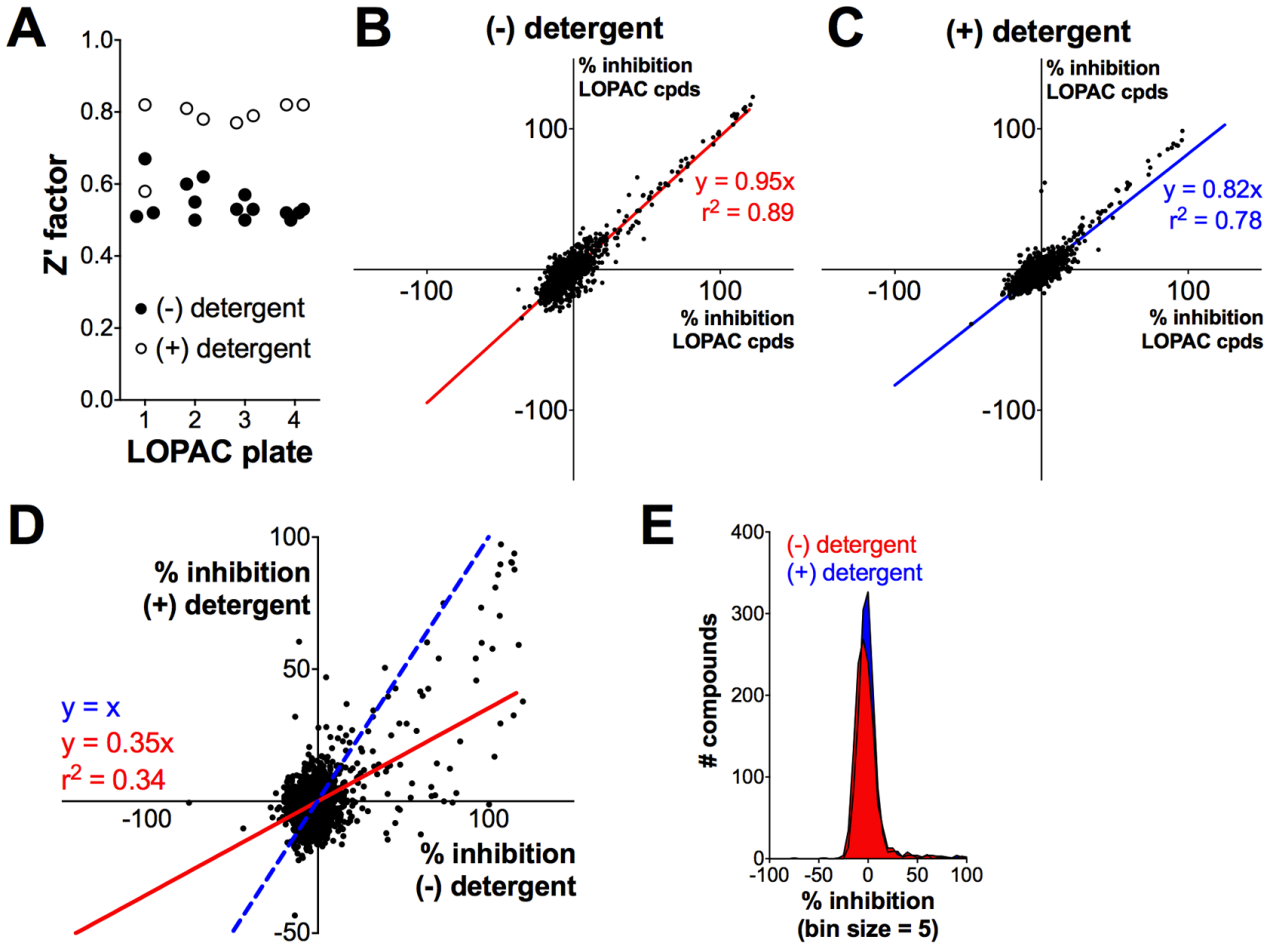
Assay validation using the LOPAC ± detergent. (A) Z' factors for replicate LOPAC experiments ± detergent. (B–C) Comparison of duplicate runs of the LOPAC ± detergent. Each point represents the activity of a discrete compound from the LOPAC. (D) Comparison of the LOPAC results ± detergent. Percent inhibitions represent the means of the replicate LOPAC experiments. Trend line (solid, red), ideal correlation line (dashed, blue). (E) Percent inhibition distribution of the averaged LOPAC results ± detergent, binned in 5% intervals.

Assay quality was evaluated using the LOPAC prior to initiating both HTS1 and HTS2. For both the first and second production runs, the LOPAC plate Z' factors were consistently above the standard cut-off value of 0.5 (Figure 3A). Duplicate runs of the LOPAC on separate days gave reproducible results for both HTS production runs (Figures 3B and 3C and Figure S1). However, the averaged LOPAC results between the first and second production runs showed less than expected correlation (Figure 3D). We suspect this may be due to the addition of the detergent in HTS2, which may alter the inhibitory behavior of compounds based on their natural propensities to aggregate. Interestingly, there was a general decrease in the percent inhibition for the most active compounds when detergent was added. Both production runs had Gaussian-like distributions centering on zero percent inhibition (Figure 3E). Likewise, there were no prohibitive row or column effects during either LOPAC experiment (Figure S2). Heat maps of each LOPAC plate from HTS1 did not reveal any prohibitive systematic errors due to well position (Figure S2). The general reproducibility of independent LOPAC experiments and their lack of any egregious systematic errors therefore suggested a full-scale HTS could be performed with similar quality.

### HTS of compound library

**Figure 4:**
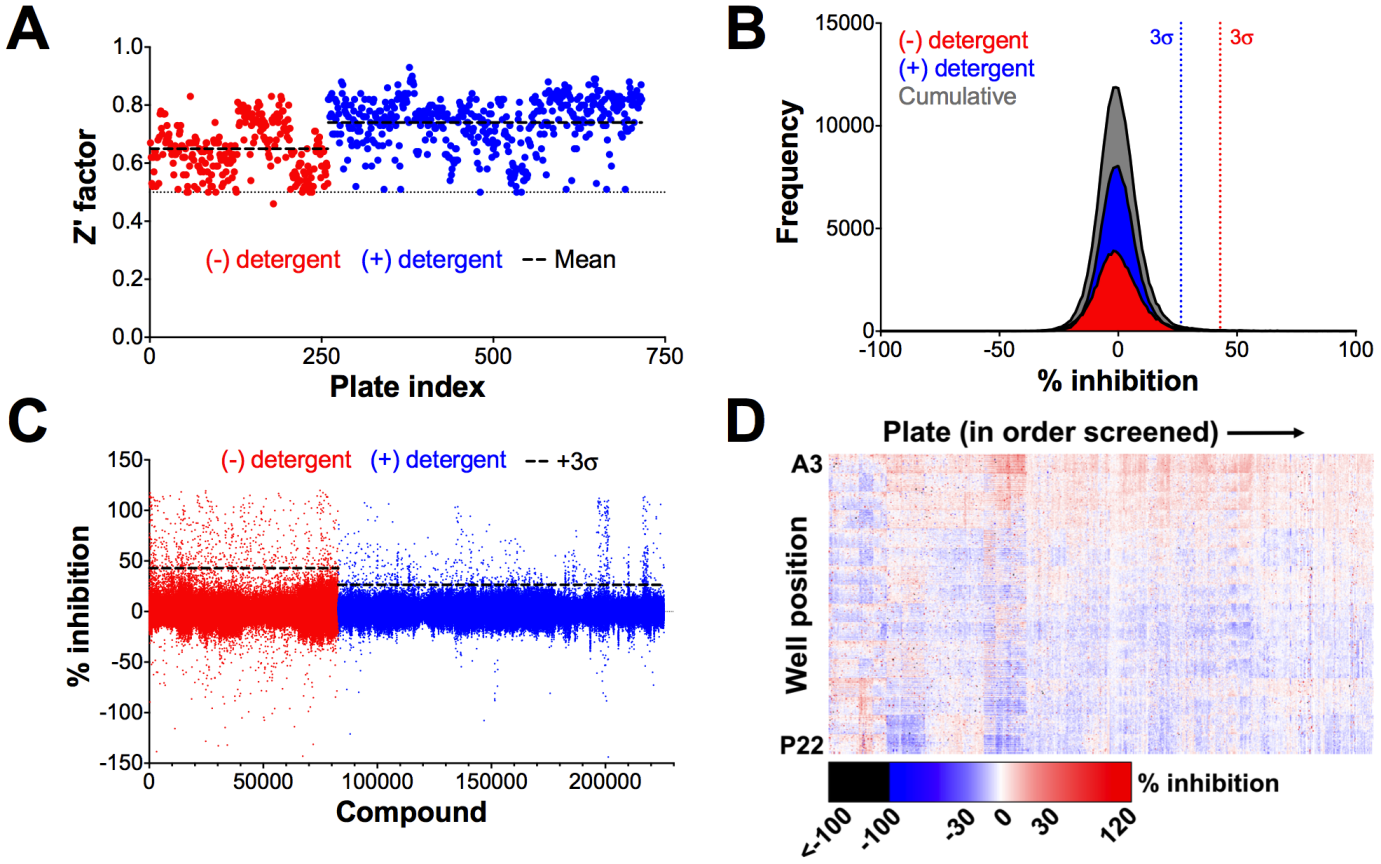
Rtt109-Vps75 HTS results. (A) Z' factors for each HTS plate, arranged in order screened. (B) Percent inhibition distribution pattern of compounds screened, binned in 1% intervals (outliers not shown). Cumulative results are pooled from HTS1 and HTS2. (C) Percent inhibition of each compound arranged in order screened. (D) Heat map of each assayed compound, arranged by well position and assay plate in order screened. HTS1 spans plates 1–259 while HTS2 spans plates 260–715. Active compounds were calculated separately for HTS1 and HTS2. HTS1 = no detergent; HTS2 = detergent.

A total of 82,861 and 142,842 compounds were screened at 10 µM final concentrations for inhibition of Rtt109-catalyzed histone acetylation in two separate production runs, respectively. The average Z' factors for HTS1 and HTS2 were 0.65 and 0.73, respectively, demonstrating that by this metric, the assay had acceptable signal dynamic range, data variation and overall quality (Figure 4A and Table 3). Only ten plates (<2%) had to be re-screened because of unacceptably low Z' factors, but these plates yielded acceptable Z' factors upon rescreening. The inhibition distributions for the HTS1 and HTS2 compounds were both normal and were both centered about zero percent inhibition (Figure 4B and Table 3). HTS2, which contained detergent in the assay buffer, had a smaller standard deviation than HTS1 (Table 3). Despite having fewer compounds screened, HTS1 contained approximately twice the number of compounds showing inhibition at several arbitrary cut-offs (Table 4). The active compounds from the primary screen - defined as those compounds having greater than three standard deviations (3σ) above the mean percent inhibition - were relatively evenly distributed in HTS1 when analyzed by screening order (Figure 4C). HTS2, on the other hand, had several prominent active clusters, such as those at approximately the 200,000th and 220,000th compounds screened (Figure 4C). Based on the chemotypes of the compounds in these clusters and the excellent corresponding Z' factors, we attribute this observation to the library organization rather than systematic errors.

**Table 3:** Rtt109-Vps75 HTS summary.

Assay	Compounds screened	Plates screened	Mean percent inhibition (%)	Mean Z' factor
HTS1 (no detergent)	82,861	259	0.00±14	0.65±0.09
HTS2 (detergent)	142,842	456	0.03±9	0.73±0.09
Cumulative (HTS1+HTS2)	225,703	715	0.02±11	0.71±0.10

**Table 4:** Activity breakdown of Rtt109-Vps75 HTS.

	Number of compounds		
Percent inhibition (%)^a^	HTS1 (no detergent)	HTS2 (detergent)	Cumulative
>95	113	46	159
90–95	24	17	41
75–90	89	39	128
50–75	287	149	436
40–50	238	148	386
30–40	575	302	877
3σ^b^	667 (0.80%)	920 (0.64%)	1587^c^ (0.70%)

a = compounds tested at 10 µM final reaction concentrations;

b = 3σ corresponds to 42.9 and 26.4 percent inhibition for HTS1 and HTS2, respectively;

c = combined from the separate HTS1 and HTS2 analyses.

The HTS was also analyzed for plate position effects as part of standard quality controls. An assay heat map depicting the inhibition of each compound as a function of well position and plate order revealed a noticeable row effect for both HTS1 and HTS2, as percent inhibition generally decreased as a function of plate row (Figure 4D and Figure S3). This effect was consistent throughout most of the assay plates for both HTS1 and HTS2. Whole-HTS analysis of the mean percent inhibition as a function of well position showed a clear row effect. In general, the mean percent inhibition decreases from the top (row A) to the bottom (row P) of the plates, whereas no such effect was discernible for the plate columns (Figure S4). We speculate this effect may be due to the orientation of the plates in the oven during the reaction. The magnitude of this row effect is approximately one standard deviation of the mean HTS inhibition. Therefore, one of our rationales for choosing a 3σ activity cut-off was to attenuate the influence of this row effect when selecting active compounds. Indeed, the row effect was less apparent when analyzing for the position of active compounds (Figure S4). Generally speaking, the above observations held true when HTS1 and HTS2 were analyzed separately (Figures S5 and S6). We also observed a checkerboard pattern in the average percent inhibition as a function of well position, though this effect was most pronounced for HTS1 (Figures S4 and S5B). We speculate the observed checkerboard pattern is due to a combination of plate orientation and our instrumentation, specifically the microplate liquid dispensing system and the microplate reader. While not performed in this study, in the interests of improving potential adaptations of our screening method, we point out that alternative statistical options are available to account for systematic trends [65], [66], [67], [68]. This may be important, for instance, when mining the primary screening data for less efficacious but potentially potent inhibitors may require additional corrections for the row effect.

### Dose-response of selected compounds

**Figure 5:**
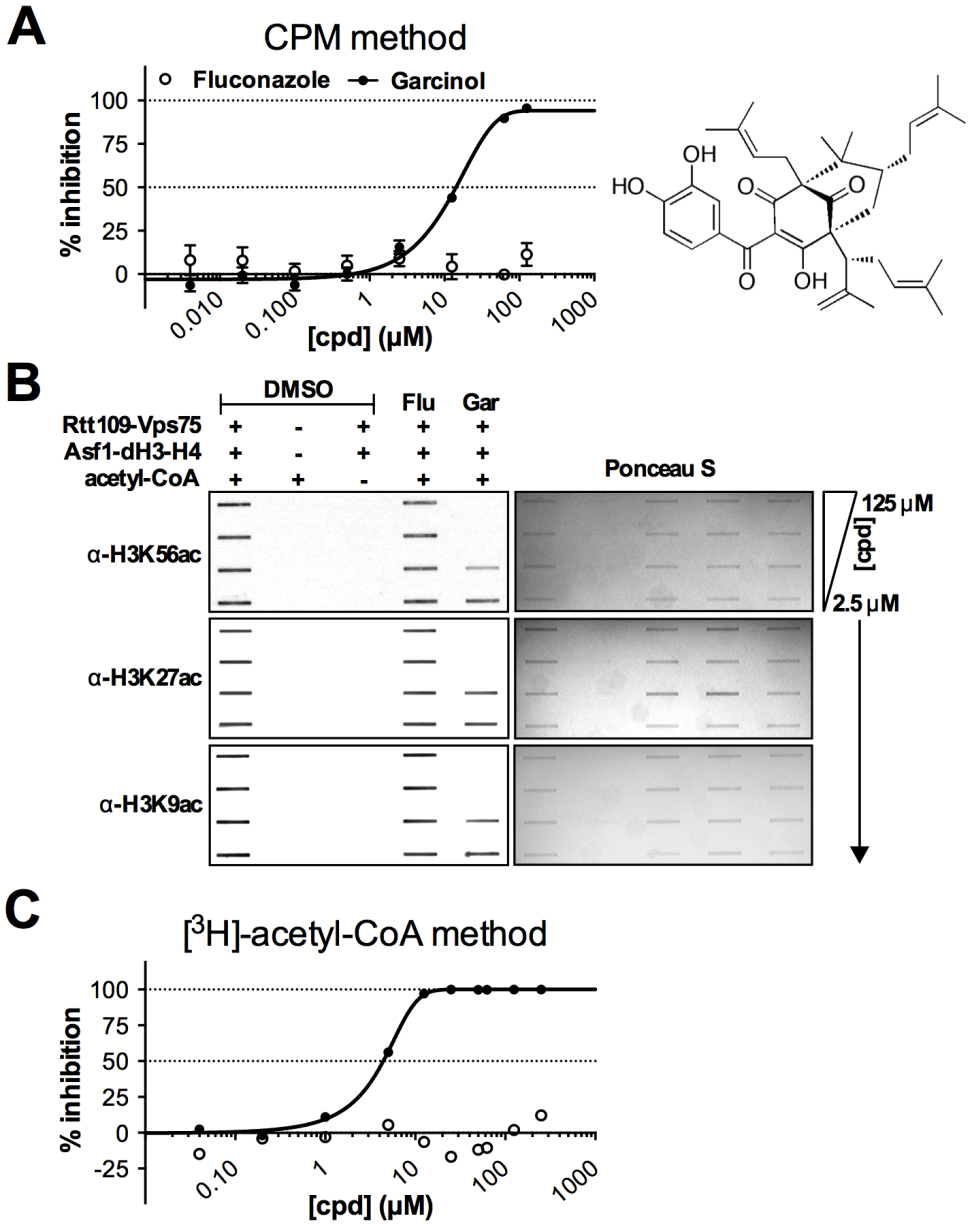
Example dose-response and orthogonal assays using garcinol, a previously reported KAT inhibitor. (A) CPM-based assay dose-response experiments with garcinol. Fluconazole = negative control. (B) Orthogonal slot blot assay to detect the presence of H3K56ac, H3K27ac and H3K9ac by Western blot. Equal protein content was verified for each membrane by Ponceau S staining. (C) Dose-response experiments with garcinol using a secondary [^3^H]-acetyl-CoA-based orthogonal assay.

To further demonstrate that this HTS can identify compounds capable of inhibiting KAT activity *in vitro*, we first present garcinol as an active compound and a previously reported KAT inhibitor that can be identified our HTS assay methods and subjected it to additional dose-response testing in the presence of detergent. Garcinol is a reported inhibitor of p300 and PCAF with IC_50_ values of 7 and 5 µM, respectively [69], [70]. The exact mechanism of inhibition by garcinol is still unclear, and it is worth pointing out that garcinol contains an *ortho*-catechol group, which flags it as a PAINS because of its potential for assay interference, potentially via an *ortho*-quinone [51], [71], [72], redox-activity [73], [74] or an oxidative product [75]. Despite these liabilities for non-specific or therapeutically uninteresting inhibition, this compound was still chosen as proof-of-concept because other reported histone acetyltransferase inhibitors were either inactive against Rtt109 by multiple assay methods or displayed prohibitive levels of assay interference (data not shown). As a negative compound control, we included fluconazole, an antifungal that inhibits the fungal cytochrome P450 enzyme 14α-demethylase with no reported activity against KATs. Garcinol, but not fluconazole, showed modest activity against Rtt109-catalyzed histone acetylation in the CPM-based assay (IC_50_ = 13 µM, Figure 5A). To confirm that garcinol decreased Rtt109-catalyzed histone acetylation, reaction aliquots were analyzed for H3K56, H3K27 and H3K9 acetylation using an orthogonal antibody-based slot blot method. This method utilizes modification-specific antibodies to detect changes in the actual histone acetylation, rather than the CoA by-product. Consistent with the CPM-based assay, garcinol showed dose-dependent decreases in H3K56ac, H3K27ac and H3K9ac when analyzed via slot blots, while fluconazole did not show any measureable decrease in histone acetylation. Equal protein content was verified by Ponceau S staining (Figure 5B).

Additional studies were performed to link the HTS assay readout to the status of acetylated histones. Garcinol showed only modest fluorescence quenching at concentrations greater than its IC_50_ value (Figure S7). Second, garcinol did not show prohibitive levels of assay interference near its IC_50_ in a CoA-based counter-screen where the acetyl-CoA substrate is replaced with CoA (Figure S8). Additionally, no adducts between garcinol and either CoA or glutathione were detected by UPLC/MS analyses of reaction aliquots under HTS-like conditions. By contrast, this assay detected several known in-house compound-CoA and compound-glutathione adducts under identical conditions (data not shown). Garcinol also did not show detectable levels of H_2_O_2_ production in HTS-like conditions in either the presence or absence of DTT (Figure S9). Garcinol showed only modest inhibition of β-lactamase activity in detergent-free, HTS-like conditions, but no such inhibition was observed when detergent was present (Figure S9). These two results strongly suggest inhibition by garcinol in our assay setup is not through redox-activity or aggregation. We further confirmed the inhibition seen in the CPM-based method by a secondary orthogonal assay that measures the amount of [^3^H]-acetate incorporated onto histones from an [^3^H]-acetyl-CoA substrate (IC_50_ = 5 µM, Figure 5C).

### Active pan-assay interference compounds

**Figure 6:**
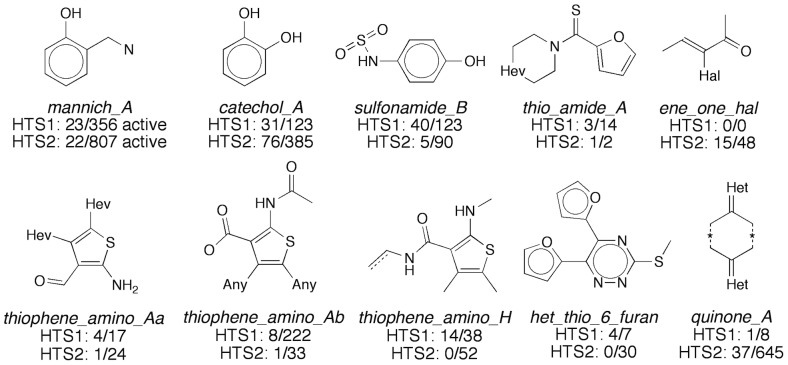
Noteworthy PAINS substructures in the primary Rtt109-Vps75 HTS. *Italics* denote the original names of published PAINS substructures [51]. For individual substructures, the ratios denote the number of primary active compounds divided by the number of compounds tested for each HTS production run.

A growing concern in academic drug discovery and high-throughput screening is the proliferation of PAINS in the literature and patent applications [76]. Based on a series of published substructures of promiscuous compounds [51], we analyzed the active compounds from the primary screen for the presence and enrichment of PAINS using cheminformatics methods (Figure 6). For HTS1, the substructure filters flagged 3,319 out of the 82,861 tested compounds as PAINS (4%), while 9,568 out of 142,842 tested compounds were flagged for HTS2 (7%). Cumulatively, the HTS library for this screen consisted of 12,887 compounds flagged as PAINS by substructure queries (6%). The frequency of each PAINS substructure in the tested compound library were in general agreement with a previous report (data not shown) [51]. For HTS1 and HTS2, the 667 and 920 active compounds contained 171 (26%) and 214 (23%) compounds that were flagged as PAINS, respectively. Both the HTS1 and HTS2 actives were especially enriched with Mannich bases, catechols and p-hydroxyarylsulfonamides. We also observed several thiophene derivatives and quinones among the actives (Figure 6).

### Post-HTS triage

**Figure 7:**
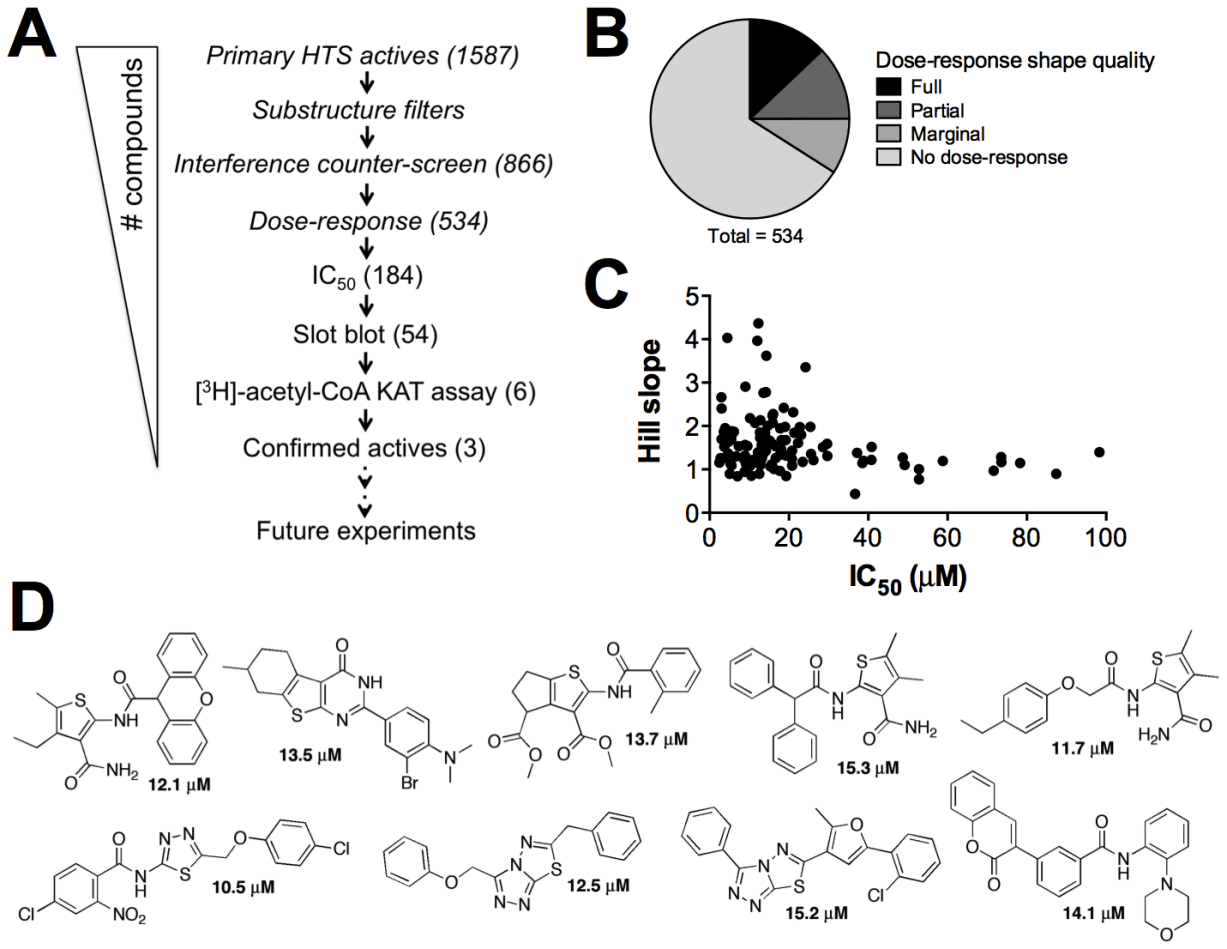
Post-HTS triage. (A) Post-HTS triage schematic. *Italics* = HTS library samples. (B) Summary of confirmatory dose-responses, as classified by shape. Full response: >95% percent inhibition span and two horizontal asymptotes; partial response: >30% span; marginal response: >15% span. (C) IC_50_ profile of purchased solid samples. (D) Chemical structures of representative artifact compounds identified by the orthogonal slot blot assay.

Given the nature of this HTS method (e.g. fluorescence-based, thiol-reactive probe, thiol-containing reaction product), we speculated a significant portion of the primary active compounds were assay artifacts. Therefore, active compounds were subjected to a series of filters, counter-screens and orthogonal assays to identify compounds that truly inhibited histone acetylation (Figure 7A). First, primary actives were computationally filtered for PAINS and other undesirable structural components such as maleimides, thiols and certain functional groups with terminal sulfur atoms. This step effectively triaged one-half of the primary screen actives. Compounds were then subjected to a counter-screen that mimicked the HTS assay conditions, except that proteins were omitted and the acetyl-CoA substrate was replaced with the CoA reaction product [41]. Given the possibility that compounds could both interfere and still inhibit enzyme-catalyzed histone acetylation, we chose to triage most compounds with greater than 50% interference. Compounds with intermediate interference levels were triaged and passed depending on several factors, including chemical diversity, the potential for compound-thiol reactivity and the interference patterns observed for each compound. Next, library compounds were assessed for their ability to generate a dose-response curve. Compounds were scored according to curve shape (Figure 7B). The overall paucity of *bona fide* HAT inhibitors reported in the literature coupled with the observation that a recent, well-designed screen versus a human HAT discovered only one true hit [59] suggested the number of true-positives in our HTS may also be relatively low. Therefore, only compounds with no observable dose-response were triaged, which eliminated more than one-half of the remaining compounds. We obtained solid powder samples for all the remaining compounds and tested them for IC_50_ confirmation. Approximately one-half of the purchased solid samples had measureable IC_50_ values, typically in the 5–25 µM range (Figure 7C). IC_50_ values were determined in two separate experiments with similar values and curve shapes, further demonstrating the assay output is reproducible (data not shown). Nearly all of these samples did not show interference in a repeat of the CoA-based counter-screen (data not shown).

**Figure 8:**
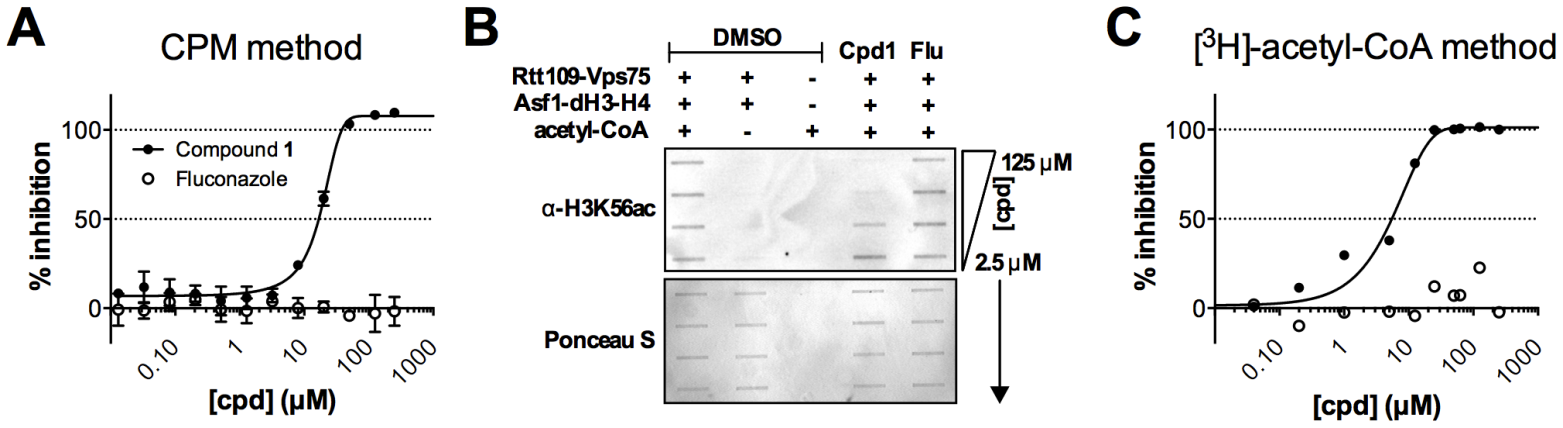
Example active compound discovered with the Rtt109 HTS and post-HTS triage. (A) CPM-based assay dose-response with compound **1**. Fluconazole = negative control compound. (B) Orthogonal slot blot assay to detect the presence of H3K56ac by Western blot. Equal protein content was verified for each membrane by Ponceau S staining. (C) Dose-response of compound **1** using a [^3^H]-acetyl-CoA orthogonal assay.

Orthogonal assays were performed next to verify if the test compounds inhibited the actual enzyme-catalyzed acetylation of histones. Based on the shape of the dose-response curves, the chemotype clusters in the confirmed actives, and other medicinal chemistry considerations, 54 compounds were tested using the orthogonal slot blot assay to probe for decreases in the actual histone acetylation. Remarkably, only six compounds tested showed definitive inhibition of histone acetylation by this method. As there is a growing interest in the chemotypes of assay artifacts, we report representative compounds that showed low micromolar IC_50_ values but were inactive in the slot blot orthogonal assay (Figure 7D). Many of these confirmed inactive compounds contained 2-amino-3-carbonyl-thiophene, 1,3,4-thiadiazole or [1,2,4]triazolo[3,4-*b*][1,3,4]thiadiazole cores. A secondary orthogonal assay that utilizes [^3^H]-acetyl-CoA confirmed only three of these remaining compounds as inhibitors of Rtt109-catalyzed histone acetylation in the low micromolar range, consistent with their CPM-based IC_50_ values (data not shown, see Figure 8).

### Identification of novel active compounds by HTS method

The CPM-based HTS method and post-HTS triage steps identified novel active compounds from a screening library that inhibit Rtt109-catalyzed histone acetylation *in vitro*. As evidence, we present compound **1**, which was identified in HTS2 as a primary active and successfully navigated the aforementioned triage process. Compound **1** showed dose-response activity when tested from library samples and re-purchased powder samples (IC_50_ = 19 µM; Figure 8A). For this compound, the orthogonal slot blot assay showed a dose-dependent decrease in H3K56ac consistent with the CPM-based HTS assay readout (Figure 8B). Compound **1** also showed activity when tested by the second, [^3^H]-acetyl-CoA orthogonal assay (IC_50_ = 4 µM, Figure 8C). Like garcinol, compound **1** showed only modest signs of fluorescence quenching at high concentrations (Figure S7). This compound did not show significant assay interference until compound concentrations above its IC_50_ value (Figure S8). However, no adducts of compound **1** with CoA or glutathione were detected by UPLC/MS analyses of aliquots reacted in HTS-like conditions. Compound **1** did not show detectable levels of H_2_O_2_ under HTS-like conditions in the presence or absence of DTT (Figure S9), strongly suggesting it is not inhibiting histone acetylation through redox activity. Similarly, compound **1** only modestly inhibited β-lactamase in the absence of detergent (Figure S9), reducing the likelihood the enzymatic inhibition is due to chemical aggregation. Neither compound **1** nor its chemical analogs have shown bioassay promiscuity according to PubChem queries (accessed 01 August 2013; data not shown). The data for compound **1** demonstrates that novel compounds identified by our method are capable inhibiting histone acetylation.

## Discussion

We have adapted previously reported CPM-based assays of CoA levels to screen 225,703 compounds for small-molecule inhibitors of the yeast KAT Rtt109. Several aspects of this cell-free assay were designed to model the Rtt109-catalyzed histone acetylation in a physiologically relevant fashion, including the use of two chaperones present *in vivo*, Asf1 and Vps75, as well as full-length histone proteins. The assay was completed in two separate, non-overlapping production runs, HTS1 and HTS2, which were performed in the absence and presence of detergent, respectively. Cumulatively, the assay had acceptable Z' factors of 0.71±0.10. Multiple independent LOPAC screens demonstrated the day-to-day reproducibility of the assay for both production runs. The cumulative primary screen hit rate was 0.70% when using a 3σ percent inhibition criterion as an activity cut-off for each production run. As proof of concept, the assay method is capable of identifying garcinol, a previously reported inhibitor of KAT activity. Follow-up studies showed garcinol causes dose-dependent inhibition of Rtt109-catalyzed histone acetylation consistent with the CPM-based assay results, though the exact mechanism of this inhibition is unclear. We also confirmed that the HTS signal readout for garcinol was not due to assay interference such as fluorescence quenching, redox-activity, or compound-thiol adducts.

Several features in our assay were meant to model potentially important protein-protein interactions involved in Rtt109-catalyzed histone acetylation in an HTS-compatible format. Specifically, we emphasize the inclusion of two purified protein complexes, the Rtt109-Vps75 enzyme complex and the Asf1-dH3-H4 substrate complex. Screening Rtt109 in the absence of Vps75 and Asf1 could presumably identify inhibitors of the Rtt109 active site. However, Rtt109 activity is significantly attenuated in the absence of its known chaperones Asf1 and Vps75 *in vitro* [4], [6], [15], [17], [33]. In contrast to most KATs that utilize free histones or nucleosomal histones as substrates, the ideal substrate for Rtt109 is the heterotrimeric complex of Asf1-dH3-H4 *in vitro* and most likely in cells, as Asf1 is essential for H3K56ac *in vivo* [4], [16]. Vps75 and Asf1 also direct the substrate specificity of Rtt109 differently [77]. Vps75 forms a complex with Rtt109 *in vivo*, and in the absence of Vps75, Rtt109 is sensitive to proteolysis [13], [19]. The choice of substrate is also a nontrivial matter. Histone substrates can vary considerably across KAT assays, including histone peptides [78], biotinylated histone peptides [59] and full-length histones. Histone precipitation can be problematic, and one solution is to use histone peptides in place of full-length histone proteins [33]. In an effort to make the assay more physiologically relevant [79], we were able to successfully incorporate full-length histone proteins complexed with the histone chaperone Asf1 into this assay. This is in contrast to the method used to identify a reported Rtt109 inhibitor, which utilized histone peptides in the primary HTS [38]. In principle, the use of both chaperones and full-length histones could allow the identification of multiple classes of inhibitors. This includes compounds that can directly target Rtt109, or disrupt the interactions between Rtt109 and Vps75, Rtt109-Vps75 and its substrate Asf1-dH3-H4, as well as those disrupting interactions between Asf1 and dH3-H4.

While this assay used the yeast Rtt109-Vps75-Asf1 system, in principle the HTS could be adapted to screen clinically relevant pathogenic fungal species such as *C. albicans* or *P. carinii*. A potential advantage of using yeast Rtt109 and its chaperones is the abundance of structural information already available (e.g. PDB IDs 2HUE, 2ZFN, 3CZ7, 3C9B, 3QM0, 3Q35, 3Q66). Additionally, inhibitors of the scRtt109 system may also inhibit Rtt109 from other fungal species, as scRtt109 can complement the function of Rtt109 from several opportunistic fungal species. For instance, it is known that *S. cerevisiae* or *C. albicans* cells lacking Rtt109 are sensitive to the chemotherapeutic agent camptothecin (CPT) [5], [34]. Importantly, expression of scRtt109 in *rtt109* ^−/−^ *C. albicans* cells renders the mutant cells resistant to CPT to a similar degree as expression of caRtt109 (data not shown). Moreover, expression of pcRtt109 in *S. cerevisiae* cells lacking scRtt109 also leads to CPT resistance and restoration of H3K56ac (data not shown). This suggests that Rtt109 from these fungal species have similar functions and that small-molecule scRtt109 inhibitors (or chemical adaptations thereof) could inhibit Rtt109 from other species, depending on the nature of the binding site(s) (i.e. the degree of sequence and structural homology) and potentially other species-specific factors.

Our assay production is also notable because of the decision to include detergent mid-assay. We decided not to re-screen the compounds assayed under non-detergent conditions based on two primary factors: resource conservation and the availability of several available follow-up assays to identify promiscuous aggregators [55], [80]. Interestingly, the mean percent inhibition, the standard deviation and the percentages of primary screen hits were lower for the detergent-containing production run (HTS2) compared to the detergent-free run (HTS1). Also, the mean Z' factor was higher for HTS2 compared to HTS1. In the absence of re-testing the same compounds under detergent and detergent-free conditions, one can only speculate these observations are related to the presence of detergent in the HTS. Other factors such as protein batches and the chemical library compositions could also account for the observed differences between HTS1 and HTS2. Based on our experience with this assay, we echo previous reports recommending the inclusion of detergents in cell-free enzymatic screens when experimentally feasible [63].

There are many reported methods for assaying acetyltransferase activities, with some being more amenable to HTS than others [81]. Radioactive filter binding assays using either [^3^H]- or [^14^C]-acetyl-CoA can be very sensitive and straightforward [82], [83], although the use of these radionucleotides on a high-throughput scale may be prohibitive. Other methods such as AlphaScreen [59] and scintillation proximity assays (SPA) have been reported for KATs [84]. These methods require specialized equipment and reagents, and in the case of SPA, special safety and waste-disposal procedures as well. Antibody-based approaches can be very specific and sensitive; however, antibodies may also bind non-specifically to undesired epitopes or possibly interfere with small-molecules resembling acetylated lysine residues, such as acetamide motifs [59]. Electrophoretic mobility shift assays have also been applied to KATs [85], but this also requires specialized instrumentation and oftentimes truncated histone substrates. CoA formation can also be assessed with a coupled enzymatic assay [57], [86], but these assays can require significant product formation for signal changes and add additional components that may be susceptible to assay interference. Advantages of our reported assay include its use of relatively standard HTS equipment, consumables and reagents. It also requires non-burdensome safety and waste-disposal procedures. Clearly, there are advantages and disadvantages to each assay method, and investigators should carefully consider the particular research question when selecting an assay.

There are also limitations to our HTS method, including the need for careful follow-up mechanism of action studies to determine the exact protein(s) inhibited in our assay. As with any HTS, we re-emphasize the need for rigorous follow-up studies to triage false-positives, assay artifacts and intractable leads [87]–[89]. Reported sources of drug discovery artifacts such as compound aggregation [56], [61]–[64], redox-active compounds [53], [54], [90], [91], non-specific protein reactivity [71], [92] and impurities [93]–[95] should be excluded by standard follow-up experiments. Compound promiscuity should also be considered [96], and compounds flagged as PAINS should be pursued with strong skepticism [76].

Our triage experience reinforces several important caveats to this CPM-based method, namely the number of apparent assay artifacts. Several aspects of the detection method make it susceptible to artifacts. First, assaying for reduction in fluorescence intensity raises the possibility for selecting compounds that are fluorescence quenchers. Second, the generation of the assay signal requires a covalent bond formation between CoA and CPM, which raises the possibility of selecting for reactive chemotypes that interfere with either reagent. Screening a large number of diverse compounds, as is routinely done in HTS, means encountering these types of artifacts is all but inevitable. However, these compounds can be triaged with appropriate control experiments [41], [52] and orthogonal assays.

The prevalence of assay artifacts therefore necessitates a rigorous post-HTS triage. Although PAINS have not been culled out from screening libraries, they can still be efficiently removed post-HTS with computational filters. Filtering specific chemotypes with well-characterized interference should also significantly enhance the triage process for thiol probe-based screens. Based on preliminary experiments, we were able to include some chemotypes like thiols and terminal sulfur atoms (e.g. certain types of thiones) in our substructure filters. Additional interfering chemotypes and the chemical nature of their interference will be reported in due course. For our particular triage, we found the slot blot format to be a convenient method to identify compounds that inhibit actual histone acetylation. In retrospect, it may be more economical to perform the slot blot or similar antibody-based histone modification assays at earlier stages of the triage process, as it may mitigate the need for interference counter-screens. Future follow-up studies on the remaining active compounds will include mechanistic and selectivity experiments. Based on the high prevalence of artifacts encountered in our primary screen actives, we highly recommend having an orthogonal assay established prior to initiating a CPM-based HTS.

Overall, the number of confirmed active compounds was relatively few. There are several non-exclusive explanations for these findings. First, this could be due to the compound library composition, as the discovery of a lead compound is predicated on the chemical matter screened. For instance, identifying a protein-protein interaction inhibitor may be difficult with traditional libraries [97]. Second, histone acetyltransferases may be inherently difficult to target with small-molecules. Several reported screens versus histone acetyltransferases have turned up few confirmed actives [38], [59]. Third, our triage process may have been overly rigorous at certain stages. Computational filtering may have removed potential actives, though many of the filtered primary actives were PAINS or thiols. It is also possible that assay interference compounds may still inhibit enzymatic activity. However, such compounds may be thiol reactive, a property that is conventionally avoided in lead discovery [71], [92]. Finally, another explanation for the low active rate observed is the HTS method employed, which may have led to false-negatives. This is difficult to assess in the absence of re-screening our library or employing an alternate screening method. However, two observations downplay this last possibility. First, our method can identify garcinol as well as novel active compounds that were confirmed by two orthogonal assays. Second, we purposefully chose a low activity threshold for IC_50_ confirmation to minimize the chance of bypassing low potency inhibitors.

To summarize, we successfully developed a CPM-based HTS to screen for inhibitors of Rtt109-catalyzed histone acetylation using two full-length physiologically relevant protein complexes. The assay is robust and straightforward, but it is susceptible to many sources of signal artifacts that fortunately can be triaged with appropriate experiments. This assay method can identify garcinol, a reported inhibitor of other KATs, though its mechanism of inhibition is unclear. Since garcinol contains a catechol, it is flagged by PAINS filters. Its activity using our HTS method confirms that active compounds identified by HTS should always be investigated with appropriate orthogonal assays, counter-screens and other follow-up experiments. This HTS identified three confirmed actives following an extensive post-HTS triage. This particular screen, or adaptations thereof, can identify compounds that inhibit Rtt109-catalyzed histone acetylation. These types of inhibitors have potential utility as chemical probes for epigenetic studies [98] as well as minimally toxic antifungals.

## Supporting Information

Figure S1 **Reproducibility of independent LOPAC experiments under detergent-free conditions (HTS1).** Shown are the comparisons of four independent LOPAC runs.
(TIFF)

Figure S2 **Plate positional effects during LOPAC experiments.** (A) Mean percent inhibition of the LOPAC ± detergent, sorted by either plate row or column. Boxes represent one standard deviation from the mean, whiskers span the 10 to 90 percentiles and dots represent outliers. (B) Heat maps of the percent inhibition results from the LOPAC plates. Each position represents the mean of the replicate LOPAC experiments ± detergent. HTS1 = no detergent; HTS2 = detergent.
(TIFF)

Figure S3 **Trellis plot of individual plate heat maps.** Plates are arranged by order screened. HTS1 spans plates 1–259, HTS2 spans plates 260–715.
(TIFF)

Figure S4 **Whole-HTS plate positional effects.** (A) Cumulative heat map showing the mean percent inhibition for each well position. (B) Cumulative mean percent inhibition and the number of actives, sorted by either row or column. Boxes represent one standard deviation from the mean, whiskers span the 10 to 90 percentiles. (C) Heat map showing the number of active compounds for each well position (greater than three standard deviations above the mean percent inhibition).
(TIFF)

Figure S5 **Analysis of HTS results in the absence of detergent (HTS1).** (A) Mean percent inhibition (top panels) and the number of actives (bottom panels; compounds with greater than three standard deviations above the mean percent inhibition for HTS1), sorted by either plate row or column. Boxes represent one standard deviation from the HTS1 mean, whiskers span the 10 to 90 percentiles. (B) Heat map showing the mean percent inhibition for each well position in HTS1. (C) Heat map showing the number of active compounds for each well position in HTS1.
(TIFF)

Figure S6 **Analysis of HTS results in the presence of detergent (HTS2).** (A) Mean percent inhibition (top panels) and the number of actives (bottom panels; compounds with greater than three standard deviations above the mean percent inhibition for HTS2), sorted by either plate row or column. Boxes represent one standard deviation from the HTS2 mean, whiskers span the 10 to 90 percentiles. (B) Heat map showing the mean percent inhibition for each well position in HTS2. (C) Heat map showing the number of active compounds for each well position in HTS2.
(TIFF)

Figure S7 **Fluorescence quenching counter-screen.** Pre-formed CPM-CoA solutions were spiked with either DMSO or test compounds. Data is expressed as the fluorescence intensity of spiked solutions relative to DMSO controls. Fluconazole = negative control compound; BHQ-1 = positive control compound.
(TIFF)

Figure S8 **Assay interference counter-screen.** Select compounds were incubated with CoA and then CPM under HTS-like conditions, minus proteins and acetyl-CoA. Fluconazole = negative control compound.
(TIFF)

Figure S9 **Redox-activity and aggregation counter-screens.** (A) Redox-activity of selected compounds using a surrogate HRP-phenol red assay. Fluconazole = negative compound control; NSC-663284 and 4-amino-1-naphthol = positive compound controls. Neither positive compound control showed detectable absorbance at 610 nm in assay buffer (data not shown). (B) Aggregation tendencies of selected compounds using a surrogate β-lactamase-nitrocefin assay. Compounds were tested at 10 µM final concentrations. Lidocaine = negative aggregation control; rottlerin = positive aggregation control. Percent inhibitions calculated based on DMSO controls.
(TIFF)
