# Supplementary figures and images for: A Cell-Free Fluorometric High-Throughput Screen for Inhibitors of Rtt109-Catalyzed Histone Acetylation
Source: PLoS One. 2013 Nov 18;8(11):e78877. doi: 10.1371/journal.pone.0078877 (PMC3832525; doi:10.1371/journal.pone.0078877)

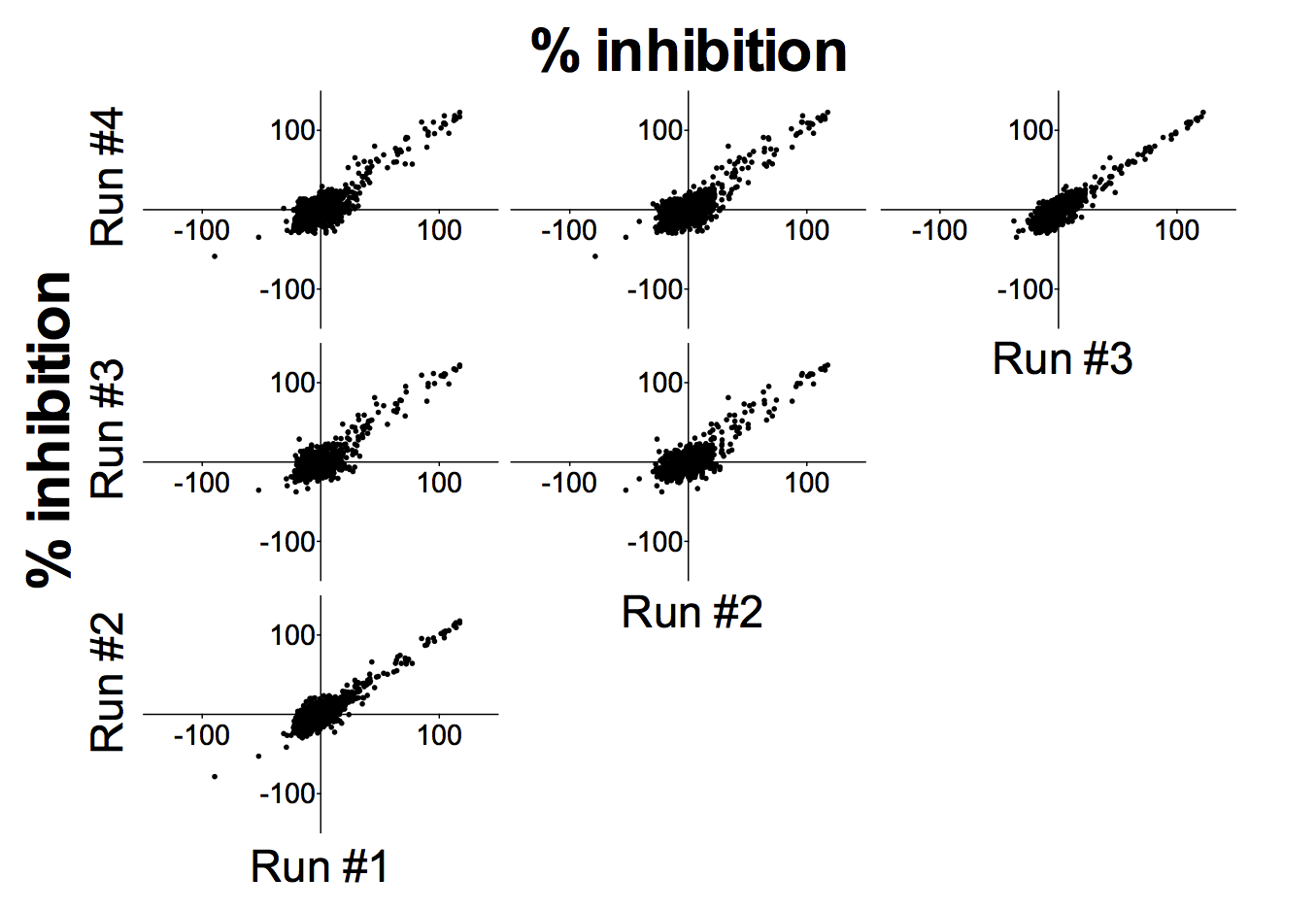

Supplement: Figure S1 — Reproducibility of independent LOPAC experiments under detergent-free conditions (HTS1). Shown are the comparisons of four independent LOPAC runs. (TIFF) [file pone.0078877.s001.tif]

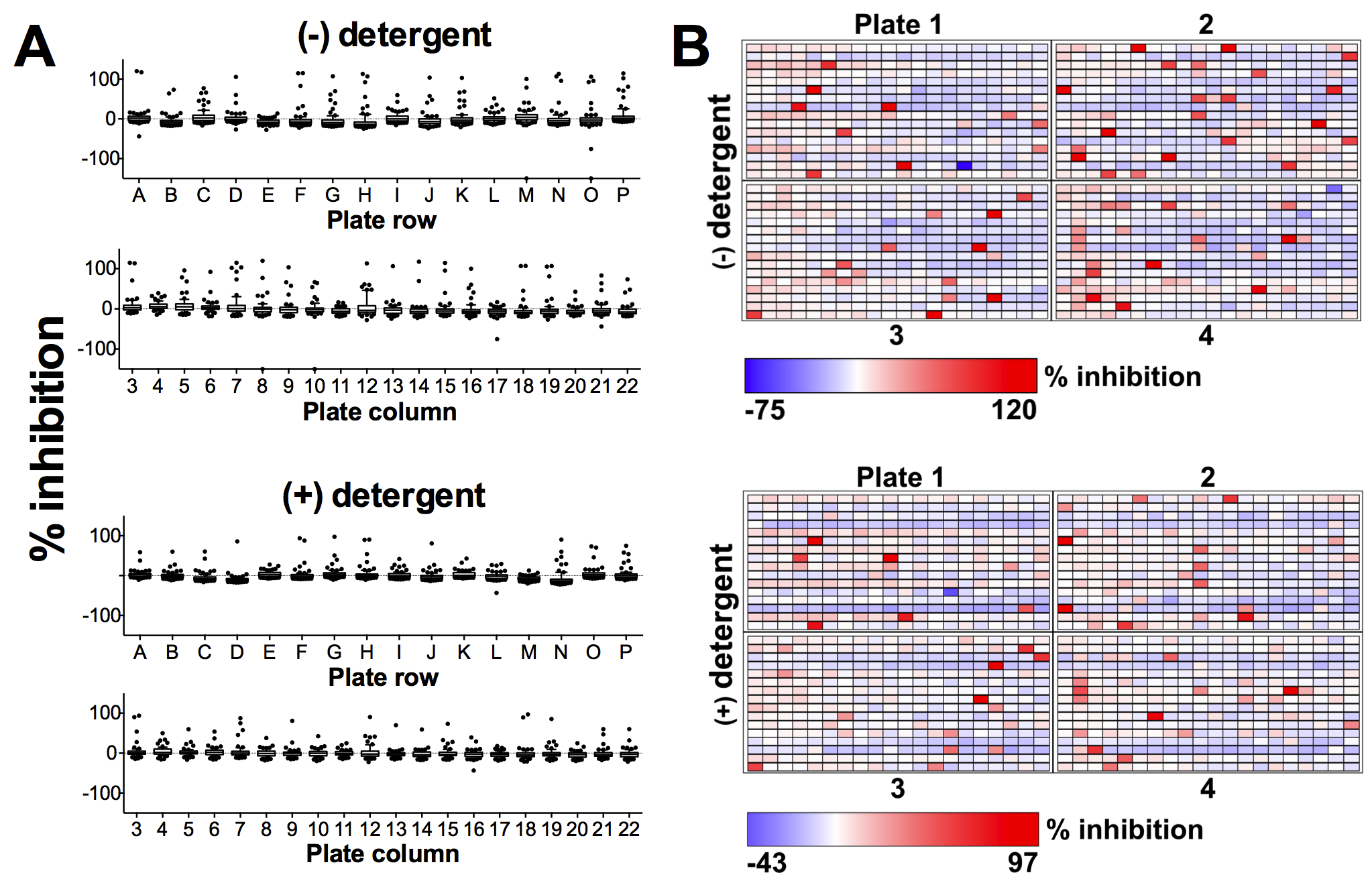

Supplement: Figure S2 — Plate positional effects during LOPAC experiments. (A) Mean percent inhibition of the LOPAC ± detergent, sorted by either plate row or column. Boxes represent one standard deviation from the mean, whiskers span the 10 to 90 percentiles and dots represent outliers. (B) Heat maps of the percent inhibition results from the LOPAC plates. Each position represents the mean of the replicate LOPAC experiments ± detergent. HTS1 = no detergent; HTS2 = detergent. (TIFF) [file pone.0078877.s002.tif]

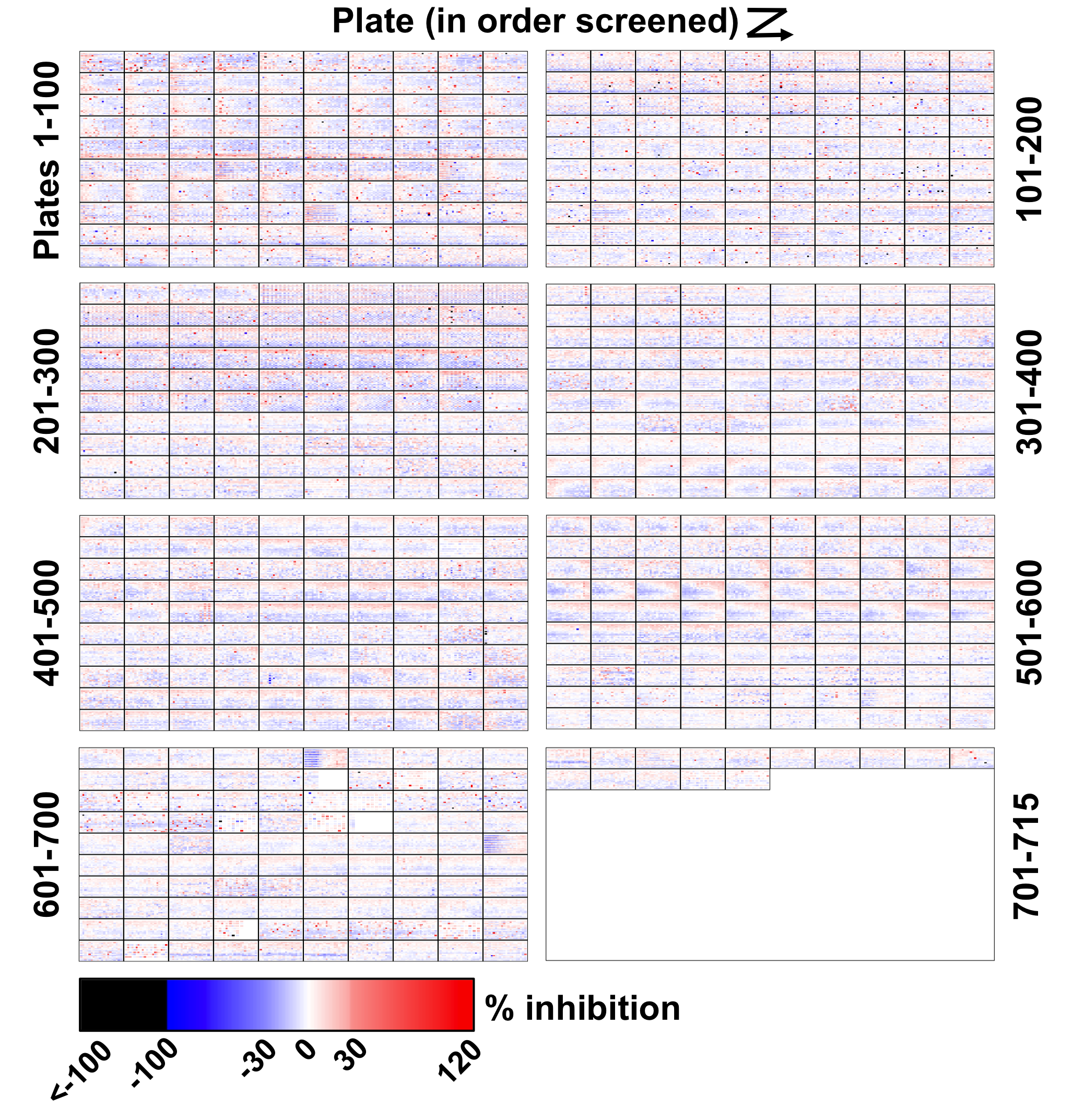

Supplement: Figure S3 — Trellis plot of individual plate heat maps. Plates are arranged by order screened. HTS1 spans plates 1–259, HTS2 spans plates 260–715. (TIFF) [file pone.0078877.s003.tif]

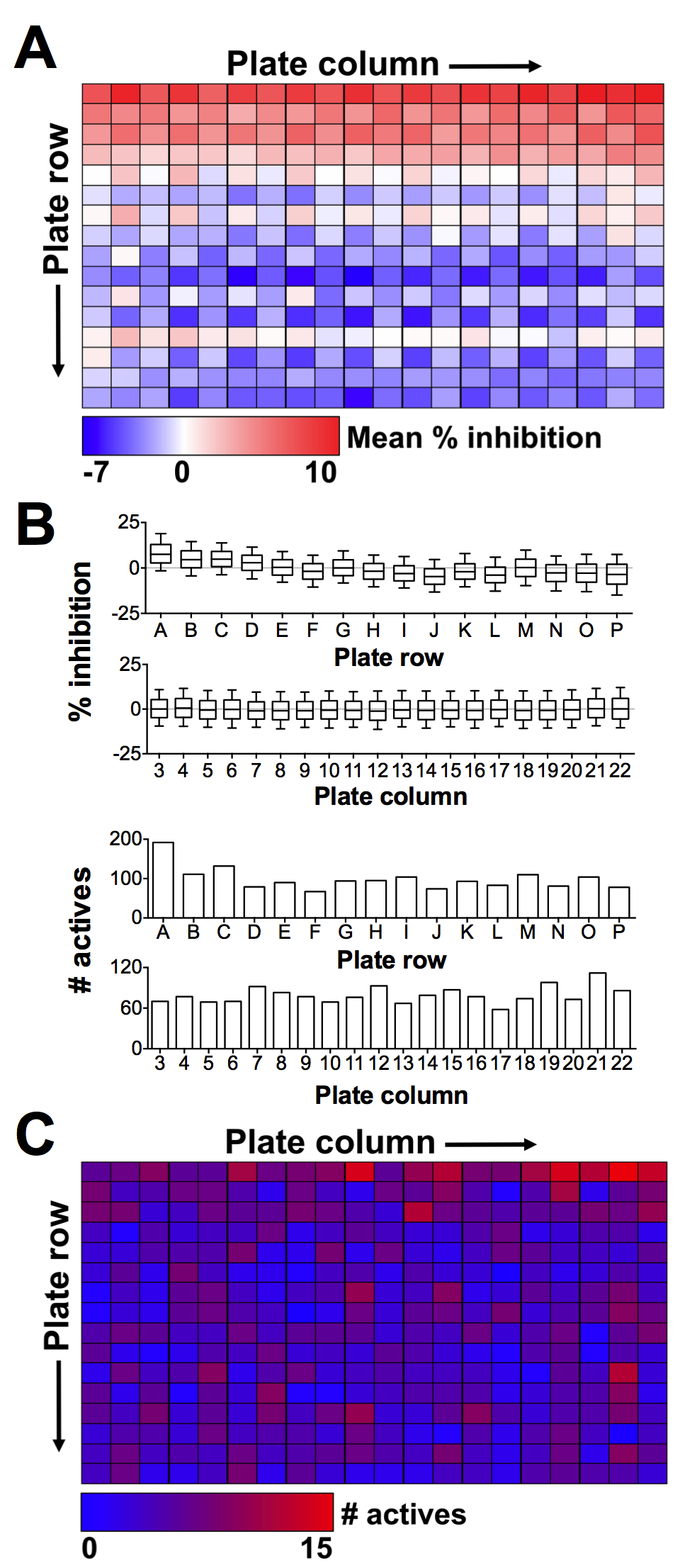

Supplement: Figure S4 — Whole-HTS plate positional effects. (A) Cumulative heat map showing the mean percent inhibition for each well position. (B) Cumulative mean percent inhibition and the number of actives, sorted by either row or column. Boxes represent one standard deviation from the mean, whiskers span the 10 to 90 percentiles. (C) Heat map showing the number of active compounds for each well position (greater than three standard deviations above the mean percent inhibition). (TIFF) [file pone.0078877.s004.tif]

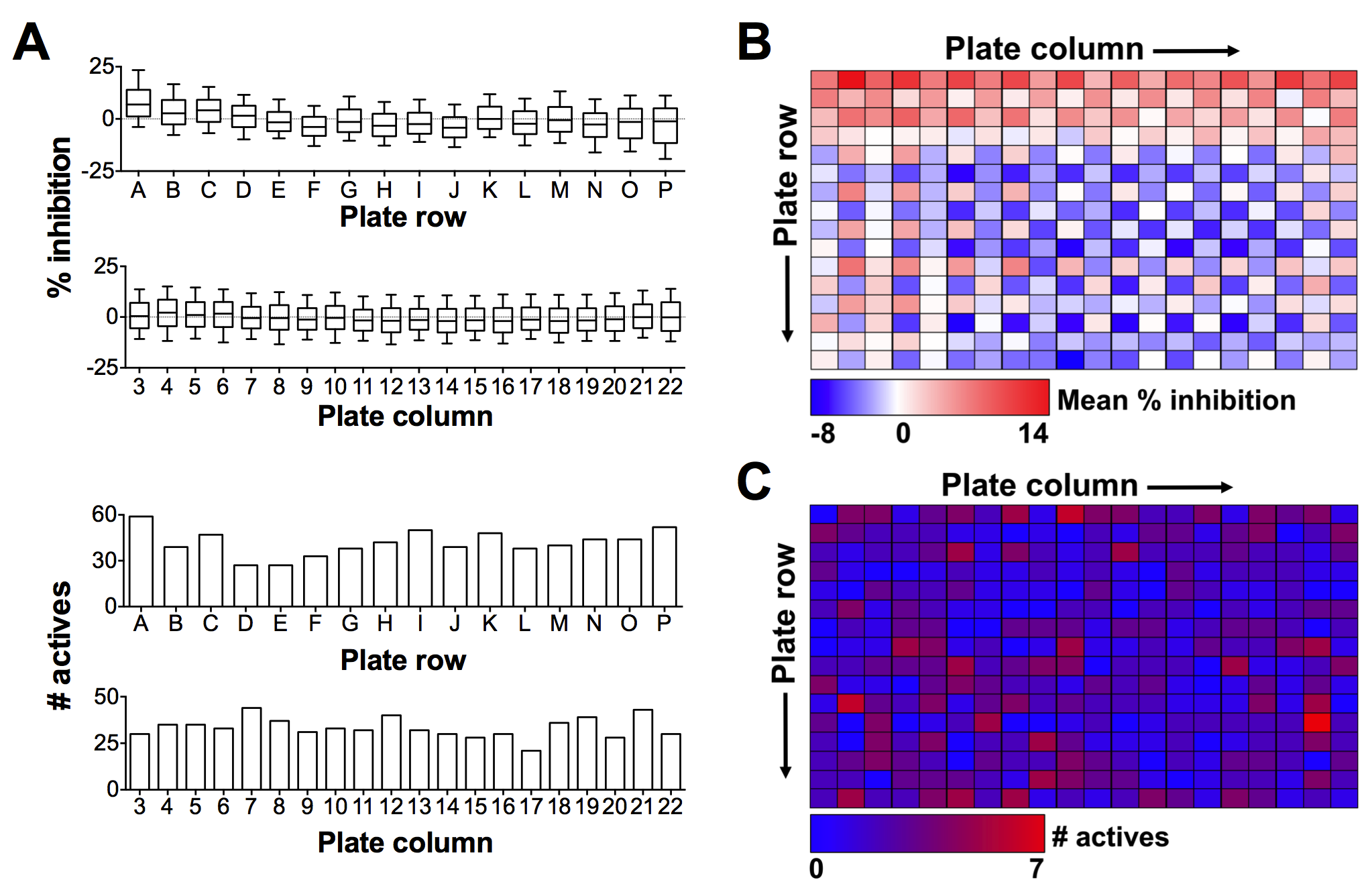

Supplement: Figure S5 — Analysis of HTS results in the absence of detergent (HTS1). (A) Mean percent inhibition (top panels) and the number of actives (bottom panels; compounds with greater than three standard deviations above the mean percent inhibition for HTS1), sorted by either plate row or column. Boxes represent one standard deviation from the HTS1 mean, whiskers span the 10 to 90 percentiles. (B) Heat map showing the mean percent inhibition for each well position in HTS1. (C) Heat map showing the number of active compounds for each well position in HTS1. (TIFF) [file pone.0078877.s005.tif]

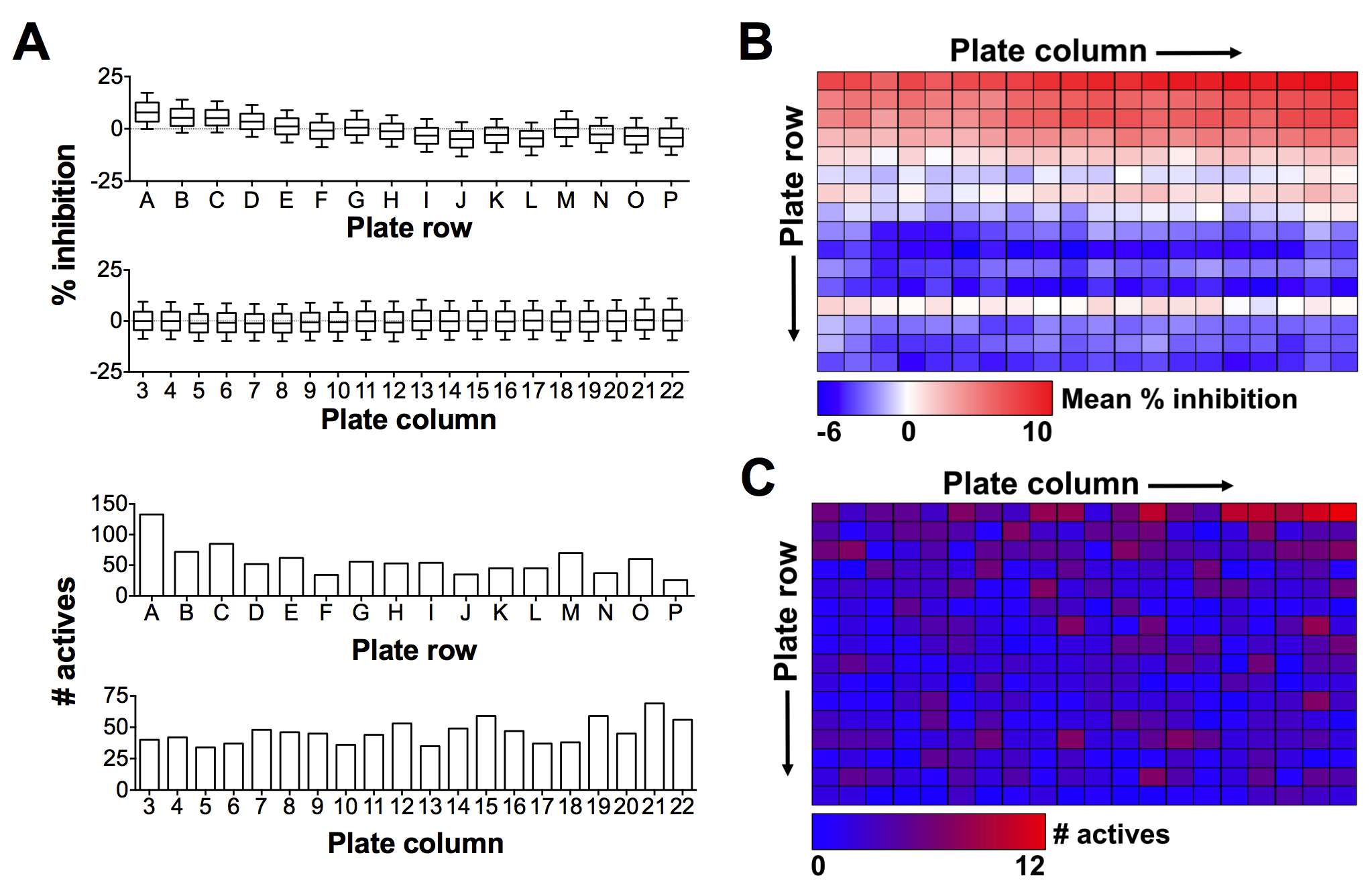

Supplement: Figure S6 — Analysis of HTS results in the presence of detergent (HTS2). (A) Mean percent inhibition (top panels) and the number of actives (bottom panels; compounds with greater than three standard deviations above the mean percent inhibition for HTS2), sorted by either plate row or column. Boxes represent one standard deviation from the HTS2 mean, whiskers span the 10 to 90 percentiles. (B) Heat map showing the mean percent inhibition for each well position in HTS2. (C) Heat map showing the number of active compounds for each well position in HTS2. (TIFF) [file pone.0078877.s006.tif]

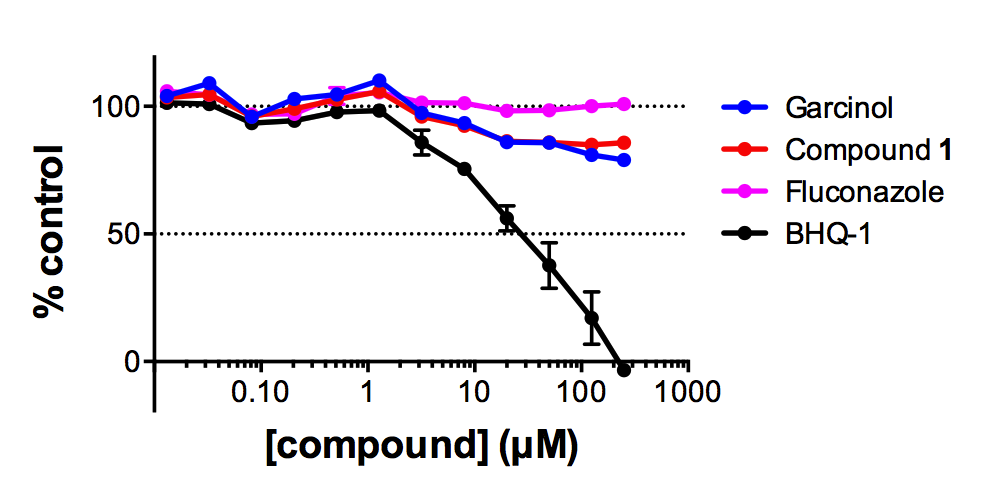

Supplement: Figure S7 — Fluorescence quenching counter-screen. Pre-formed CPM-CoA solutions were spiked with either DMSO or test compounds. Data is expressed as the fluorescence intensity of spiked solutions relative to DMSO controls. Fluconazole = negative control compound; BHQ-1 = positive control compound. (TIFF) [file pone.0078877.s007.tif]

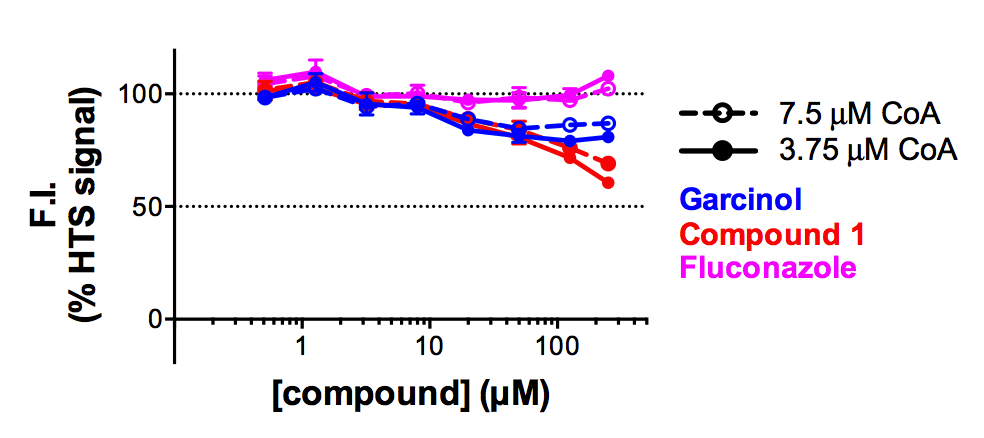

Supplement: Figure S8 — Assay interference counter-screen. Select compounds were incubated with CoA and then CPM under HTS-like conditions, minus proteins and acetyl-CoA. Fluconazole = negative control compound. (TIFF) [file pone.0078877.s008.tif]

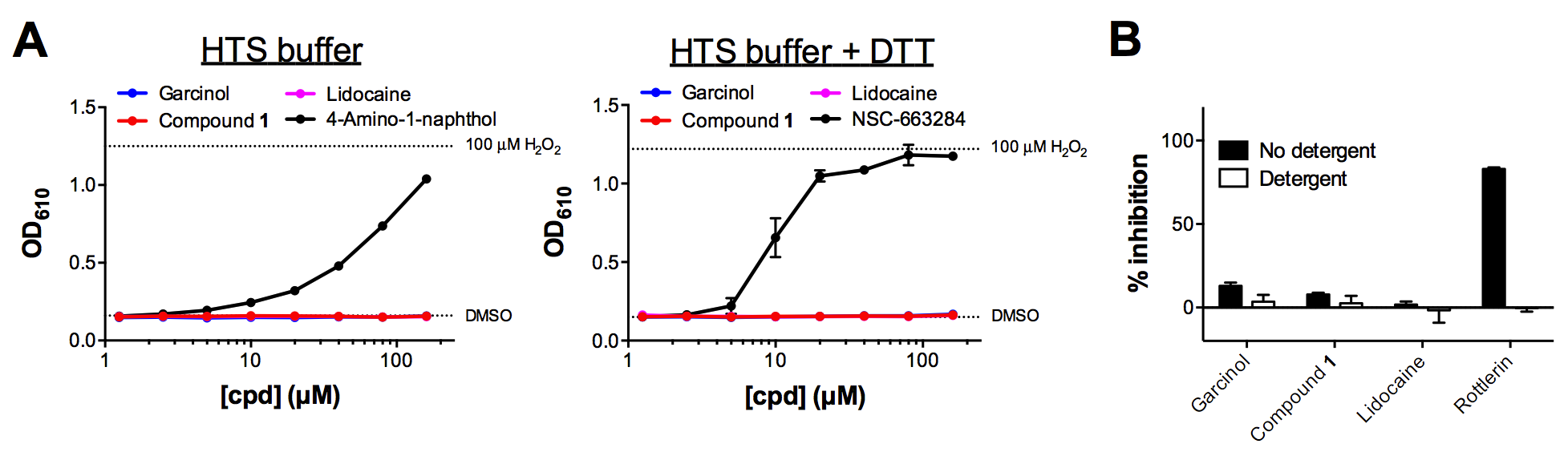

Supplement: Figure S9 — Redox-activity and aggregation counter-screens. (A) Redox-activity of selected compounds using a surrogate HRP-phenol red assay. Fluconazole = negative compound control; NSC-663284 and 4-amino-1-naphthol = positive compound controls. Neither positive compound control showed detectable absorbance at 610 nm in assay buffer (data not shown). (B) Aggregation tendencies of selected compounds using a surrogate β-lactamase-nitrocefin assay. Compounds were tested at 10 µM final concentrations. Lidocaine = negative aggregation control; rottlerin = positive aggregation control. Percent inhibitions calculated based on DMSO controls. (TIFF) [file pone.0078877.s009.tif]
